# In vitro cultured malaria hypnozoites leave a footprint of specific metabolites

**DOI:** 10.1371/journal.ppat.1013577

**Published:** 2025-10-16

**Authors:** Erica M. Pasini, Hassan Hakimi, Nattawat Chaiyawong, Onny Klop, Anne-Marie Zeeman, Ivonne Nieuwenhuis, Nicole van der Werff, Kumiko Kihara, Kenjiro Kami, Clemens H. M. Kocken, Osamu Kaneko

**Affiliations:** 1 Biomedical Primate Research Center, Department of Parasitology, Rijswijk, The Netherlands; 2 Department of Protozoology, Institute of Tropical Medicine (NEKKEN), Nagasaki University, Nagasaki, Japan; 3 National Institute of technology, Kumamoto college, Kumamoto, Japan; 4 Human Metabolome Techonologies, Inc., Tsuruoka, Yamagata, Japan; University of Geneva Faculty of Medicine: Universite de Geneve Faculte de Medecine, SWITZERLAND

## Abstract

*Plasmodium vivax* malaria control and elimination is complicated by the presence of dormant liver stages, known as hypnozoites, that can reactivate weeks, months or years after infection giving rise to clinical and transmissible *vivax* malaria, without exposure to new infectious mosquito bites. Hypnozoite infection remains without symptoms and there are no diagnostic tools available to identify hypnozoite carriers. Such diagnostic tools are invaluable for precise mapping of the scale of the infection problem and for identifying individuals that would qualify for targeted drug treatment, to wipe out this hidden reservoir of malaria parasites. Targeted treatment would have considerable benefits as it would prevent the exposure of individuals without hypnozoites to the considerable side-effects of drugs such as Primaquine, which has a relatively high toxicity to people deficient in glucose-6-phosphate dehydrogenase. Here we present a Proof-of-Concept study aimed at identifying diagnostic markers for malaria hypnozoite infection, by combining *in vitro Plasmodium cynomolgi* hypnozoite cultures (an accessible proxy to *P. vivax* with nearly identical biology) with sensitive metabolomics. Specific hypnozoite-related metabolites have been identified in the supernatant of hypnozoite-enriched *in vitro* liver stage cultures. This suggests that, following *in vivo* validation of such metabolites in the *P. cynomolgi*/rhesus monkey model and subsequent in *P. vivax*-infected individuals, a rapid diagnostic test for hypnozoite infection may be developed.

## Author summary

Malaria caused by *Plasmodium vivax* is especially hard to eliminate because the parasite can hide in the liver as “sleeping” forms called hypnozoites. These dormant parasites can wake up weeks, months, or even years after the original infection and cause the disease to return—without any new mosquito bite. The problem is that people carrying these hidden parasites show no symptoms, and there are currently no tests available to detect them. This makes it difficult to know who needs treatment and leads to unnecessary drug use, which can cause serious side effects in some individuals. In this study, we explored a new way to detect hypnozoite infections by searching for chemical fingerprints—called metabolites—that are either released by these dormant parasites directly or by the liver as a result of their presence. Using a closely related malaria parasite (*Plasmodium cynomolgi*) grown in lab conditions, we identified specific metabolites linked to the presence of hypnozoites. This is the first step toward developing a simple test that could detect hidden malaria infections in people. If successful, such a test could improve how we treat and control *P. vivax* malaria, making it safer and more targeted.

## Introduction

Malaria continues to raise serious health concerns across the globe. While clinical malaria cases in the Asia-Pacific and the Americas have gone down >90% in the last decade, a shift in malaria species composition has been observed, with *Plasmodium vivax* representing 53% of the global burden in South-East Asia and being the predominant species in the Americas [1]. Due to its unique biology, *P. vivax* poses unique challenges to achieving the WHO ambitious targets for malaria control and eradication [2]. Asymptomatic dormant liver stage (hypnozoite) infections form a hidden parasite reservoir in the human population that can give rise to new symptomatic and transmissible malaria episodes weeks, months or years after primary infection, without new infection through mosquito bites. In light of the paradigm shift from malaria control to eradication, which has been enshrined in the United Nations Sustainable Development Goals, effective diagnostics able to discern between hypnozoite carriers and hypnozoite non-carriers as a result of a *P. vivax* malaria infection are indispensable to appropriately target drug therapies. While their development is considered a priority by the WHO, such diagnostic tools are currently lacking [2]. The availability of such tools would make it possible to map the scale of the problem by determining how many people are infected with hypnozoites world-wide in the short-term and to target drug treatment specifically to hypnozoite-carriers in the long-term. This approach of identifying and treating individuals infected with dormant malaria parasites would prevent *P. vivax* malaria transmission and disease relapse while also preventing unnecessary drug exposure of individuals not carrying hypnozoites to antimalarial drugs with considerable side-effects such as Primaquine, which has a relatively high toxicity in G6PD-deficient patients. Over a thousand transcripts are reported to be significantly differently expressed in total liver RNA from mice infected with 5 x 10^4^ rodent malaria sporozoites as compared to control mice [3], thus indicating that a malaria liver infection, with relatively few hepatocytes infected (compared to total hepatocytes in the liver), is measurable at the molecular level. Based on this, we hypothesize that dormant *P. vivax* malaria hypnozoite infections in the liver of infected individuals may be measurable in the periphery through the detection of hypnozoite- or infected liver-specific metabolites. A similar approach has successfully identified metabolites of a plant aminolevulinic acid pathway derived from *Plasmodium* blood-stage parasites in malaria patients [4].

As a first step in exploring the feasibility of developing diagnostic tools, we here apply sensitive metabolomic analysis to *P. cynomolgi* hypnozoite-enriched *in vitro* culture supernatants of *P. cynomolgi* liver stage cultures (Zeeman et al. Antimicrob Agents Chemother 2014, 2016). We use this zoonotic non-human primate malaria as it is the *P. vivax* sister parasite (Joyner et al., Front Microbiol 2015), with nearly identical biology, including hypnozoite formation. In addition, *P. vivax* parasites can only be obtained from patients in endemic areas and specific, often protected, new world monkey species, which is cumbersome, while *P. cynomolgi* parasites can be obtained from its infected natural host, the rhesus monkey, which is experimentally more readily available. The study was specifically designed to determine whether it is possible to detect differential metabolic signatures between hypnozoite-forming parasite *P. cynomolgi* and hypnozoite non-forming parasite *P. knowlesi* in a high sensitivity setting to be used as biomarkers after validation in *in vivo* pre-clinical studies. Moreover, the system is designed to replicate liver infections, which are considered asymptomatic and not to include a blood-infection component.

## Materials and methods

### Ethics statement

#### Study approval and ethics.

Non-human primates were used because no other models (in vitro or in vivo) were suitable for the aims of this project. The local independent ethical committee constituted conform Dutch law (Biomedical Primate Research Center Dier Experimenten Commissie, DEC) approved the research protocol (agreement number DEC# 751B) prior to the start and the experiments were all performed according to Dutch and European laws. The Council of the Association for Assessment and Accreditation of Laboratory Animal Care (AAALAC International) has awarded BPRC full accreditation. Thus, BPRC is fully compliant with the international demands on animal studies and welfare as set forth by the European Council Directive 2010/63/EU, and Convention ETS 123, including the revised Appendix A as well as the ‘Standard for Humane Care and Use of Laboratory Animals by Foreign Institutions’ identification number A5539-01, provided by the Department of Health and Human Services of the United States of America’s National Institutes of Health (NIH) and Dutch implementing legislation. The rhesus monkeys (male Indian *Macaca mulatta*, age 6–15 years) used in this study were captive-bred and socially housed. Animal housing was according to international guidelines for non-human primate care and use. Besides their standard feeding regime, and drinking water *ad libitum* via an automatic watering system, the animals followed an environmental enrichment program in which, next to permanent and rotating non-food enrichment, an item of food-enrichment was offered to the macaques daily. All animals were monitored daily for health and discomfort. All intravenous injections and large blood collections were performed under ketamine sedation, and all efforts were made to minimize suffering.

### Non-human primate infections

*P. cynomolgi* M strain blood stage parasites originally provided by Dr. Bill Collins from the Center for Disease Control, Atlanta, USA [5] and *P. knowlesi* H strain blood stage parasite stocks were thawed and resuspended in PBS for intravenous injection (10^6^ parasites in 1 mL of PBS for *P. cynomolgi* [6] and 10^5^ parasites in 1 mL of PBS for *P. knowlesi*) into a donor monkey. Parasitemia was monitored by thigh prick, heparin blood was collected at peak parasitemia (±1% trophozoites), and the monkey was cured from malaria by chloroquine treatment (intramuscular injection, 7.5 mg/kg, on 3 consecutive days). The collected blood was used for the *ex-vivo* feeding of mosquitoes.

### Obtaining sporozoites for in vitro hepatocyte culture infection

*P. cynomolgi* as a hypnozoite-forming parasite and *P. knowlesi* as a hypnozoite-non-forming control were used throughout the study.

To obtain *P. cynomolgi* and *P. knowlesi* sporozoites for the infection of *in vitro* cultured rhesus monkey hepatocyte cultures to initiate liver stage parasite cultures, *Anopheles stephensi* (for *P. cynomolgi*) and *An. dirus* (for *P. knowlesi*) mosquitoes were fed using membrane feeding on infected blood derived either from *P. cynomolgi*- or *P. knowlesi*-infected rhesus macaques (donor monkeys) [7]. *An. stephensi* adult mosquitoes (strain Sind-Kasur) were obtained from the Radboud University in Nijmegen, while *An. dirus* eggs were obtained from the CDC and reared locally. Sporozoites were isolated from the salivary glands of *An. stephensi* and *An. dirus* mosquitoes 15 days after feeding and used as detailed below.

### Biological and technical replicates

Overall, the experimental plan included 3 biological replicates for primary *in vitro* hepatocyte infections with the hypnozoite-forming parasite *P. cynomolgi* and 1 biological replicate for infections with the non-hypnozoite forming *vivax*-type control parasite, *P. knowlesi*, and uninfected hepatocyte controls. The primary hepatocytes were freshly isolated and plated [7] from the livers of three different rhesus macaques for the three biological replicates of the *P. cynomolgi* infection and the single *P. knowlesi* replicate. Three technical replicates (3 single wells) per time point and condition were analysed to assess the quality of the culture and the effect of the drugs and control for reproducibility. All technical replicates showed a high level of reproducibility. Thus, the 3 single wells pertaining to each specific time point and condition were pooled to obtain enough material for the metabolomics analysis. The three *P. cynomolgi* and the one *P. knowlesi* biological replicates were analyzed with capillary electrophoresis time-of-flight mass spectrometry (CE-TOFMS) platforms.

### *In vitro* liver stage culture methodology

**Fig 1:**
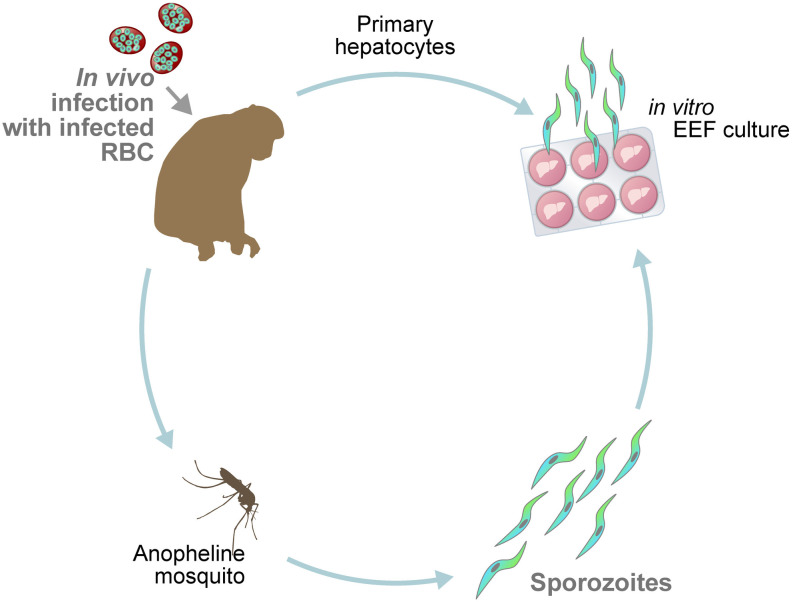
Production of sporozoites and in vitro liver culture infection. Rhesus macaques (1) are infected with blood stage parasites (A) of *P. cynomolgi* or P*. knowlesi*. Blood is drawn to feed mosquitoes and harvest the sporozoites (B) used in the in vitro infection of cultured rhesus hepatocytes (2). Upon infection of primary hepatocytes, sporozoites give rise to developing liver stages and hypnozoites in *P. cynomolgi* and developing liver stages only in *P. knowlesi*. Primary hepatocytes used in in vitro cultures are freshly harvested from an independent rhesus macaque each time (three biological replicates).

The system used for the *in vitro* liver cultures (Fig 1) represents an adaptation to the needs of the metabolomics analysis of the system first described in Zeeman et al. [7,8].

As described below, to find the ideal culture conditions for the downstream metabolomics analysis pilot experiments were carried out, which allowed for the testing of different culture conditions (e.g., different medium composition, amount of medium, frequency of medium refreshment and sporozoite inoculum). The following optimized conditions were finally used to carry out the metabolomics measurements. Briefly, primary rhesus monkey hepatocytes were isolated from liver lobes, as described by Guguen-Guillouzo et al. [9]. Ninety-six-well tissue culture plates were seeded with 70,000 freshly isolated primary rhesus monkey hepatocytes/well in 100 µL of Will-B medium (William’s E with glutamax containing 10% human serum (A+), 1% MEM nonessential amino acids solution (100x, Invitrogen), 2% penicillin/streptomycin, 1% insulin/transferrin/selenium solution (100 nM, Invitrogen), 1% sodium pyruvate solution (100x, Invitrogen), 50 μM β-mercapto-ethanol, and 0.05 μM hydrocortisone); cultures were kept at 37°C in 5% CO_2_. On day 2, the medium was changed from 100 µL to 40 µL/well and subsequently changed in four days intervals. For example, at day 2 the medium was reduced from 100 µL to 40 µL/well and left untouched until day 6 when samples were harvested for metabolomics analysis. Thus, the results of the metabolomic analysis cover days 2–6 in this case. Experimental wells were infected with 100,000 *P. cynomolgi* or 66,000 *P. knowlesi* sporozoites/well and treated identically to uninfected control wells. *P. cynomolgi*-infected liver stage cultures were carried out in three experimental conditions: standard (infected, untreated) (STD); treated with 1 µM Atovaquone between day 0–6 (ATQ; Merck Life Science N. V.), which kills developing liver stages but not hypnozoites; and treated with 1 µM phosphatidylinositol 4-kinase inhibitor (PI4Ki) between day 1–6 (PI4Ki; [10]), which which also kills developing liver stages through a different mechanism. In each case the uninfected culture served as a control for the specific condition (STD, ATQ or PI4K treated) during the metabolomics analysis. *P. knowlesi* liver infections were carried out in two culture conditions only (STD and PI4Ki). Also, in the case of *P. knowlesi*, uninfected culture served as a control for the specific condition (STD and PI4Ki) during the metabolomics analysis. The culture supernatants for metabolomics analysis were harvested on days 3, 6, 9 and 11 (d3, d6, d9 and d11; representing the accumulation of metabolites between days 2–3, 2–6, 5–9 and 7–11) for *P. cynomolgi* and on days 3, 6 and 9 for *P. knowlesi*. As *P. knowlesi* has a shorter liver cycle (5–6 days and no hypnozoites), sampling at day 11 would not yield additional information over day 9. Collected supernatants were filtered through 5-kDa filters provided by Human Metabolome Technologies, Inc. (HMT; Tsuruoka, Yamagata, Japan) to eliminate macromolecules prior to metabolomics analysis. Cells were fixed with paraformaldehyde (4%) at days 3, 6, 9 and 11 for *P. cynomolgi* and at days 6 and 9 for *P. knowlesi*. Intracellular *P. cynomolgi* and *P. knowlesi* liver stages were stained with the cross-reactive rabbit polyclonal anti-*P. cynomolgi* Hsp70 serum [7] and Alexa Fluor 647-labeled goat anti-rabbit IgG [6] (Thermo Fisher Scientific, 1:1000); 2 µM DAPI (Thermo Fisher Scientific) was used to stain the nuclei. Morphological examinations of the cells were conducted using an inverted microscope to confirm the cells’ viability. Analysis of the cultures to define their parasite composition was carried out using an automated Operetta high-content imager (Operetta; Perkin-Elmer) and where needed confirmed by visual inspection and manual counting.

### Metabolomics and statistical analysis

Metabolomics of *in vitro* culture supernatants was performed at HMT with CE-TOFMS platforms to capture a wide variety of metabolites including unknowns. Briefly, the cultured medium was centrifuged to remove debris, after which 80 µL of the supernatant was mixed with 20 µL of Milli-Q water containing internal standards (H3304-1002; HMT). The mixture was centrifugally filtered through a 5-kDa cutoff filter (ULTRAFREE MC PLHCC, HMT) at 9,100 × g, 4ºC to remove macromolecules. The filtrate was then used for metabolome analysis at HMT.

Metabolome analysis was conducted according to HMT’s Premium Plan package using CE-TOFMS based on the methods described previously [11,12]. Briefly, CE-TOFMS analysis was carried out using an Agilent CE capillary electrophoresis system equipped with an Agilent 6230 time-of-flight mass spectrometer (Agilent Technologies, Inc., Santa Clara, CA, USA). The systems were controlled by Agilent MassHunter Workstation Data Acquisition (Agilent Technologies) and connected by a fused silica capillary (50 μm i.d. × 80 cm total length) with commercial electrophoresis buffer (H3301-1001 and I3302-1023 for cation and anion analyses, respectively, HMT) as the electrolyte. The spectrometer was scanned from m/z 50–1,000 and peaks were extracted using MasterHands, automatic integration software (Keio University, Tsuruoka, Yamagata, Japan) in order to obtain peak information including m/z, peak area, and migration time (MT) [13]. Signal peaks corresponding to isotopomers, adduct ions, and other product ions of known metabolites were excluded, and the remaining peaks were annotated according to HMT’s metabolite database based on their m/z values and MTs. In addition, putative metabolites were assigned from the Human Metabolome Database (HMDB), PubChem database, and HMT peptide list on the basis of theoretical m/z calculated from a molecular formula. Areas of the annotated peaks were then normalized to internal standards and sample amount in order to obtain relative levels of each metabolite.

Statistical significance was evaluated using Welch’s t -test, resulting in a specific metabolite lists of non-infected rhesus monkey hepatocytes, hypnozoite- and developing liver stage-infected hepatocytes.

### Comparison between groups

The final metabolomics lists for each treatment in which metabolite concentrations were expressed as a ratio against the control condition after normalization were compared with one another to identify the hypnozoite-associated metabolites. PI4Ki [8] and ATQ treated cultures [14] result in an enrichment in hypnozoites and thus in their metabolic products. STD cultures after day 9 also result in a progressive enrichment in hypnozoites because growing liver exoerythrocytic forms (EEF) burst and release merozoites, leaving behind the sleeping liver stages.

### Identification of metabolites with unknown chemical structure

Initially, data from all the biological replicates under analysis were integrated and a unique ID for each peak was attributed that was consistent across replicates. Subsequently, a batch analysis of all samples was performed using CE high-resolution MS (CE-HRMS; [15]) focusing on selected unknown metabolites. The CE-HRMS analysis allowed for the matching of each of the 11 peaks across all samples in terms of their order and timing of appearance. Only metabolite peaks matching between samples were selected for further analysis. For such metabolite peaks a chemical formula prediction by CE-HRMS analysis was performed at HMT with ω Search Plan, where the prediction of candidate structures was based on precursor metabolite search, MT prediction, and MS/MS analysis. The prediction of the chemical formula was obtained through isotopic patterns extracted based on m/z and relMT from CE-HRMS data. Precursor metabolites were searched with a program called “Michi-taro” [16], while MS fragment analysis was performed by comparing the signals detected in the MS/MS analysis with the fragment patterns predicted by MS-FINDER [17].

## Results

### Optimization of the culture conditions for metabolomics analysis

**Fig 2:**
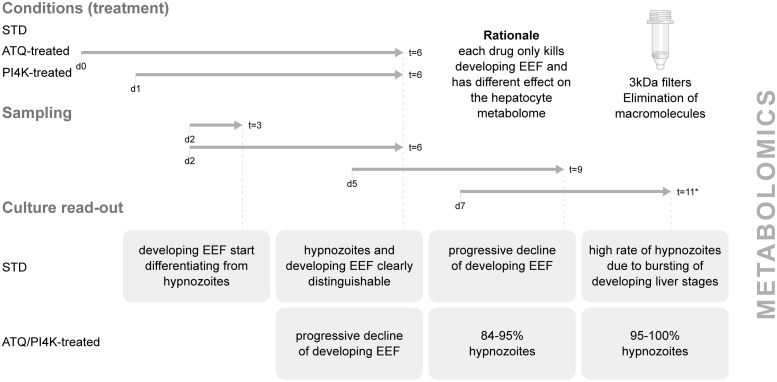
Experimental set-up for metabolomics. Samples for metabolomics were obtained from *P. cynomolgi* or P*. knowlesi* infected and uninfected *in vitro* liver cultures. Parasites were cultures in three conditions: standard (no drug treatment), Atovaquone (ATQ) and PI4K-treated. The treatment with Atovaquone started at d0 after sporozoite infection, while the treatment with PI4K started at day 1. Cultures were treated for 5 consecutive days. The rationale for using two drugs is briefly presented. The sampling strategy is shown in parallel with the culture read-out at the time of sampling. The downstream workup of the samples is shown prior to the metabolomics.

To optimize the culture system first described in Zeeman et al. [7] and render it best compatible with the metabolomics analysis, a set of pilot experiments was carried out to define key *in vitro* liver stage culture parameters. The liver stage cultures under consideration were assessed in three different conditions: infected/uninfected under untreated STD conditions and infected/uninfected treated with either ATQ or PI4Ki. As the metabolomics technology at hand uses a direct injection coupled with the CE-TOFMS methodology, it was considered important to reduce the culture volume to increase the metabolites’ concentration in the supernatant. However, the reduction of the culture volume had to be in line with the preservation of the viability of the liver stage culture in the three different conditions under consideration. The following parameters were evaluated: number of sporozoites/well; use of culture medium with/without serum; frequency with which the medium was refreshed and quantity of medium/well. The parameters used as a read-out in all the pilot experiments were the viability of the cultured cells assessed through the morphological evaluation using an inverted microscope and the increased number of metabolites detected in the supernatant assessed through pilot metabolomics studies. After different conditions were evaluated, the conditions presented in the methods were preferred. The serum-containing medium Will-B was preferred to serum-free medium because it allowed for the cultivation of healthy uninfected/infected hepatocytes even when they were cultured in a small medium volume of 40 µL/well. The health of the culture did not deteriorate during exposure to anti-malarial drug treatment with ATQ and PI4Ki, while the smaller culture volume allowed for an increase in the number of detected metabolites. In line with existing publications [6,8,14] untreated cultures infected with either *P. cynomolgi* or *P. knowlesi* showed developing EEFs until they burst. ATQ- and PI4Ki-treated cultures on the other hand showed growing EEFs only at early time points (day 3), which progressively disappeared due to the drug treatment [6,8,14]. Thus, by day 6, drug-treated cultures were enriched with hypnozoites in the case of *P. cynomolgi* (Fig 2) and devoid of living parasites in the case of *P. knowlesi*.

Good infection rates (S1 and S2 Figs and S1 Table) were observed both in untreated STD *P. cynomolgi* and *P. knowlesi in vitro* liver cultures. For both parasite species, the technical replicates yielded reproducible EEF counts (S1 and S2 Figs and S1 Table). High numbers of *P. cynomolgi* developing EEFs were detected (S1 and S2 Figs and S1 Table) in all parasite-infected cultures (drug-treated or not) at day 3, which progressively declined by day 6 in drug-treated cultures and by day 9 in untreated cultures, due to the bursting of developing EEFs. Drug treatment efficiently eliminated growing EEFs and enriched the culture for hypnozoites. No significant differences were observed between ATQ- and PI4Ki-treated cultures in the number of hypnozoites after treatment. This was in line with expectations [8]. Limited differences between biological replicates were observed whereby biological replicate two had the overall highest rate of infection. In stained *P. knowlesi in vitro* liver cultures developing liver stages were visible on day 6 in untreated cultures (STD), which progressively disappeared by day 9, due to the bursting of developing EEFs. Untreated *P. knowlesi*-infected liver cultures at day 9 show the near-to-total absence of parasite remnants. In PI4Ki-treated cultures, high numbers of developing EEFs can be seen at day 3, which are killed off efficiently by day 6.

### Metabolites significantly up-regulated between a specific culture condition and its control

Overall, between 567 and 653 metabolites were identified in the three biological replicates of the *P. cynomolgi* liver stage culture supernatants (Tables 1 and S2).

**Table 1:** Total metabolites detected by category in the three *P. cynomolgi*-infected biological replicated and in the single *P. knowlesi-*infected replicate.

	*P. cynomolgi*			*P. knowlesi*
Metabolite	Replicate 1	Replicate 2	Replicate 3	Replicate 1
Total identified	603	567	653	513
Significant up-regulated	218	170	202	162
Significant up-regulated with known chemical structure	73	66	81	71
Significant up-regulated with unknown chemical structure	145	104	121	91

**Fig 3:**
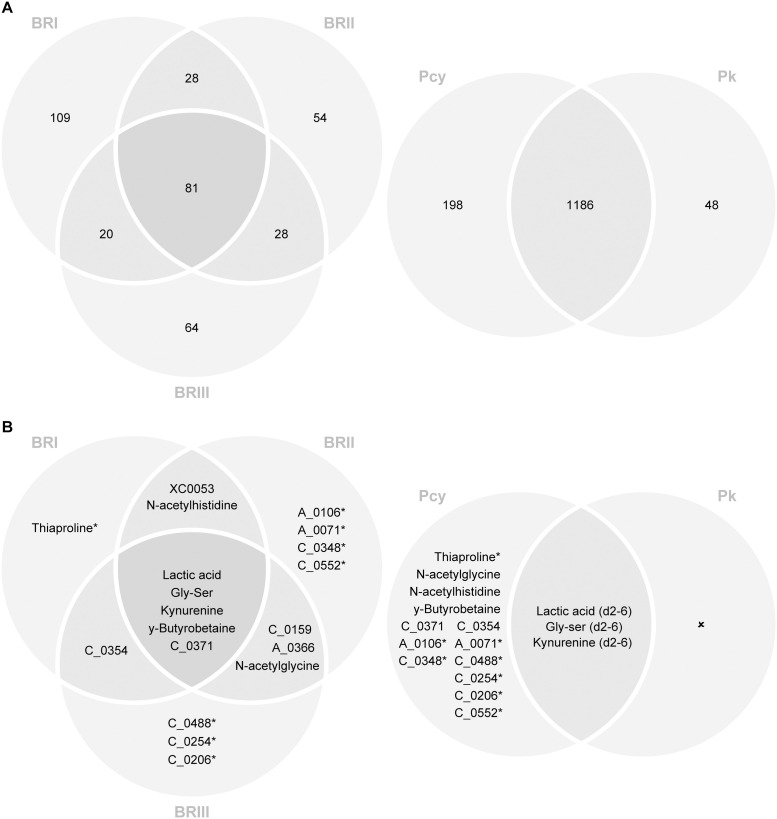
**(A)** Venn diagram of significantly up- or down-regulated metabolites. A Venn diagram of the number of significantly up- or down-regulated metabolites detected in the different biological replicates is presented. A comparison between significantly up- or down-regulated metabolites in *P. cynomolgi* samples and in *P. knowlesi* samples is also shown. **(B)** Venn diagram of the selected potentially hypnozoite-infection specific metabolites. A Venn diagram of the selected potentially hypnozoite-infection specific metabolites is presented showing in which biological replicates they were found. Some selected metabolites (*), while being found only in one biological replicate have been detected multiple times and were found to be always statistically significant or highly statistically significant at late timepoints indicative of the enrichment with hypnozoites in the cultures.

Of these metabolites, between 215 and 384 unique metabolites were significantly up-regulated in *P. cynomolgi*-infected *in vitro* liver stage cultures at one or more specific time points and in one or more of the three culture conditions (STD, ATQ-treated, PI4Ki treated) than uninfected cultures kept in identical conditions. Between 80 and 97 unique metabolites have been attributed to known chemical structures and between 88 and 126 unique metabolites are yet to be attributed (Fig 3A). Moreover, 513 unique metabolites were identified in *P. knowlesi*-infected *in vitro* liver stage culture supernatants (Tables 1 and S2). Of these metabolites, 234 unique metabolites were significantly up-regulated in *P. knowlesi*-infected *in vitro* liver stage cultures at one or more specific time points and in one or more of the two culture conditions (STD and PI4Ki). Eighty-nine could be attributed to known metabolites and 73 remain to be attributed (Fig 3A). One hundred and ten unique metabolites, for which standards were available at HMT, were quantified each time. Of the 215–384 unique significantly up-regulated metabolites detected in *P. cynomolgi* and the 234 significantly up-regulated metabolites detected in *P. knowlesi*, 186 metabolites were common to both parasites (Fig 3A).

### Hypnozoite-associated candidate metabolites

In line with Fig 2, candidate metabolites were considered hypnozoite-associated (deriving directly from hypnozoites or from changes in the liver metabolome induced by the presence of hypnozoites) when the following criteria were met:

1^st^ criterion (first priority): Metabolites that are significantly up-regulated in both ATQ and PI4Ki-treated samples at d9 (covering metabolites between day 5 and 9) or d11 (d11; covering metabolites between day 7 and 11) independently of their identification in STD conditions and absent or detected only early and not significantly up-regulated in the *P. knowlesi* metabolome.

2^nd^ criterion (second priority): Metabolites that are present in both STD and ATQ conditions at d11 or in both STD and PI4Ki at d11 and absent or detected only early and not significantly up-regulated in the *P. knowlesi* metabolome.

3^rd^ criterion (third priority): Metabolites that are identified in both STD and ATQ at d9 or in both STD and PI4Ki conditions at d9 and absent in the *P. knowlesi* metabolome; metabolites that are identified in either STD d9 or d11 and ATQ at d9 or d11 (excluding metabolites falling under the second priority) or in STD d9 or d11 and PI4K at d9 or d11 (excluding metabolites falling under the second priority) and absent in the *P. knowlesi* metabolome.

Depending on the stringency of the criteria applied to the candidate metabolites’ selection, the candidate metabolites were assigned to different priority categories. A list of the candidate metabolites detected meeting each criterion is provided in Table 2 and Fig 3B.

**Table 2:** Metabolites likely to be hypnozite infection specific.

Metabolite	1^st^ criterion	2^nd^ criterion	3^rd^ criterion
Significantly up/down-regulated with known chemical structure	*N*-acetylglycine	Gly-Ser	Kynurenine
	*N*-acetylhistidine	XC0053	
	Lactic acid	y-Butyrobetaine	
	Thiaproline		
Significantly up/down-regulatedwith unknown chemical structure	A_0106	C_0206	C_0371
	A_0071	A_0366	C_0159
	C_0348		C_0254
	C_0552		C_0488
			C_0354

### Malaria infection-related metabolites

Overall, 186 metabolites were detected, which are significantly up-regulated in both *P. cynomolgi* and *P. knowlesi*-infected *in vitro* liver stage culture supernatants. These metabolites are considered malaria (including hypnozoite) infection-specific. Eighty-one of these metabolites have been unequivocally identified, while the chemical structure of 105 remains to be ascertained.

Twenty-seven significantly up-regulated candidate metabolites have been identified that are specific for the *P. cynomolgi* hepatocyte infection and absent or detected only early and not significantly up-regulated in the *P. knowlesi* metabolome. Of these, 4 metabolites of known chemical structure are potentially hypnozoite specific (S3 Table), where lactic acid is significantly up-regulated in all three biological replicates, *N*-acetylglycine and *N*-acetylhistidine are significantly up-regulated in two biological replicates and Thiaproline is significantly up-regulated at multiple time points in one biological replicate (Table 3). Together with 4 additional candidate metabolites of unknown chemical structure, which also fit the 1^st^ criterion (S3 Table), overall, 8 candidate metabolites were detected, which can be considered priority one metabolites. A further 5 metabolites were detected, which specifically meet the 2^nd^ criterion (S3 Table). Of these 5 metabolites, 3 pertain to known chemical structures (Gly-Ser, y-Butyrobetaine and XC0053) and 2 to unknown chemical structures. A further 6 metabolites can be considered as fitting the 3^rd^ criterion (S3 Table), which include Kynurenine and 5 metabolites of unknown structure. As shown in Table 3, metabolites meeting the 2^nd^ and 3^rd^ criterion are either identified across different biological replicates or detected at multiple time points and in the different conditions under consideration within one replicate.

**Table 3:** Detection of potentially hypnozoite-associated candidate metabolites across replicates.

	*P. cynomolgi*			*P. knowlesi*
Metabolite	Replicate 1	Replicate 2	Replicate 3	Replicate 1
**1**^**st**^ **criterion**				
*N*-acetylglycine		(+)	(+)	
*N*-acetylhistidine	(+)	(+)		
Lactic acid	(+)	(+)	(+)	d2-6
Thiaproline	(+)*			
A_0106		(+)*		
A_0071		(+)*		
C_0348		(+)*		
C_0552		(+)*		
**2**^**nd**^ **criterion**				
Gly-Ser	(+)	(+)	(+)	d2-6
XC0053	(+)	(+)		
y-Butyrobetaine	(+)	(+)	(+)	
C_0206			(+)*	
A_0366		(+)	(+)	
**3**^**rd**^ **criterion**				
Kynurenine	(+)	(+)	(+)	d2-6
C_0371	(+)	(+)	(+)	
C_0159		(+)	(+)	
C_0254			(+)*	
C_0488			(+)*	
C_0354	(+)		(+)	

* multiple detections within one replicate and statistically significant

Nine metabolites that were *P. cynomolgi*-specific (identified in *P. cynomolgi* STD, ATQ- and PI4Ki-treated conditions, but not in *P. knowlesi*) and not belonging to the previous categories (e.g., detected at early time points (d2-3; d2-6) but not at later time points d5-9; d7-11)) were detected, which may be interesting as general markers of *P. cynomolgi* (and possibly *P. vivax*) hepatocyte infection. Unique metabolites specific for the *P. knowlesi* hepatocyte infection were also detected. Twelve metabolites were attributed to known IDs, while 35 are yet to be attributed. Although interesting, at present the latter metabolites will not be pursued further.

Forty-seven metabolites were detected which were either *P. knowlesi*-infection specific or significantly up-regulated only in *P. knowlesi* liver infection, whereby 12 have been attributed to known chemical structures (S4 Table) and 35 are yet to be structurally defined. Interestingly, there are also some metabolites (e.g., glycerophosphocholine) that are significantly up-regulated at multiple time points in the *P. knowlesi* hepatocyte infection, while being only sparsely significantly up-regulated across the 3 hepatocyte biological replicates infected with *P. cynomolgi*.

### Identification of potentially hypnozoite-associated candidate metabolites with unknown chemical structure

An additional effort was made to identify the unknown metabolites, which met the criteria for being potentially hypnozoite-infection specific in our final prioritized metabolite list.

Initially, 11 unknown peaks were selected (Table 2) and analyzed by CE-HRMS. Only peaks matching between biological replicate samples in terms of their order and timing of appearance were selected for further analysis. Seven peaks passed this initial screening. Namely: C_0348, C_0552, C_206, C_371, C_159, C_0254, and C_0354. Using a chemical formula prediction by CE-HRMS analysis, the structure of each of the compounds was predicted (Table 4). Two candidate structures identified for the metabolite IDs (C_0254 and C_159) are referenced in the Human Metabolome Database (HMDB). Known reference standards are available for 3 structures identified (C_0552, C_0254 and C_159) known, which can be purchased from various vendors. To identify the remaining 4 metabolites, reference compounds need to be synthesized. Such standards and/or synthetic reference compounds can be used to definitively confirm that the candidate structures predicted do indeed correspond to the specific metabolites by isotopic patterns, MT, and MS/MS fragment comparison. We have purchased the three commercially available reference standard and were able to confirm two predicted structures C_0254 and C_159 (S1 Data: CE-MS/MS analysis report) out of three. Unfotunately, it was not possible to confirm the identity of C_0552 and further biochemical characterization of this compount will need to be carried after appropriate reference compounds are synthesized.

**Table 4:** Predicted structures for seven candidate metabolites initially lacking identification.

Peak number	Predicted structure of candidate metabolite
C_0348	(2R)-3-(2-aminoethyldisulfanyl)-2-(oxaloamino)propanoic acid
C_0552	N-[2-[4-(1,1-dioxothiolan-3-yl)piperazin-1-yl]-2-oxoethyl]-1,3-benzodioxole-5-carboxamide
C_0371	3-[(2-acetamido-2-carboxyethyl)disulfanyl]-2-aminopropanoic acid
C_0206	2-ethenyl-2-oxopyrrolidine-1-sulfonamide
C_0254	2-aminoethyl 2,3-dihydroxypropyl hydrogen phosphate
C_0354	2-amino-3-[(2-amino-2-carboxyethyl)trisulfanyl]propanoic acid
C_0159	4,5,6,7-tetrahydro-3H-imidazo[4,5-c]pyridine- 6-carboxylic acid

## Discussion and conclusion

No diagnostic tools are currently available to identify malaria hypnozoite carriers. Thus, hypnozoite infections remain often undetected due to the lack of symptoms and they represent a hidden reservoir of malaria parasites, complicating malaria eradication. A first step towards a new diagnostic approach to identify hypnozoite carriers after *P. vivax* infection, based on sensitive metabolomics is presented here based on sensitive metabolomics. The availability of such a tool would prove invaluable for determining the scale of the hypnozoite infection problem and for combating this hidden parasite reservoir by specifically treating hypnozoite carriers with hypnozoite-killing drugs such as Primaquine. Targeted treatment would have considerable benefits as it would prevent the exposure of individuals without hypnozoites to the considerable side-effects of drugs such as Primaquine, which has a relatively high toxicity in G6PD-deficient patients.

A study by Na et al. [18] comparing *P. falciparum*- and *P. vivax*-infected individuals showed that metabolomic changes can be revealed in the plasma of malaria-infected patients [18]. Additional studies on *P. vivax* malaria-infected individuals and *P. cynomolgi* infected macaques have also been carried out. In a randomized control trial, Gardinassi et al. [19] have found that platelet activation-related metabolites were enriched in sick, blood-stage positive individuals following primary infections with malaria parasites [19]. Another study by Gardinassi et al. [20] shows that the abundance of some plasma metabolites varies according to the levels of parasitemia in *P. vivax* malaria patients. [20]. In a study on recurrent (newly detectable episode of blood-stage parasitemia due to hypnozoite reactivation) *P. vivax* malaria, an enrichment in the metabolic pathways related to butanoate, aspartate and asparagine, and N-glycan biosynthesis was found in the detected episodes of blood-stage parasitemia due to hypnozoite reactivation, showing that different metabolic pathways are enriched in recurrent malaria compared to the primary infection [21]. Results by Uppal et al. [22] on patients infected with chloroquine-resistant and chloroquine-sensitive *P. vivax* blood-stage parasites suggest differences in lipid, amino acids, and nucleotide metabolic pathways in the plasma of these two groups prior to antimalarial treatment [22]. Sengupta et al. [23] used NMR to define the urinary metabolome of *P. vivax*-infected patients with fever and found that *P. vivax* malaria infection is characterised by an enrichment in ornithine and pipecolic acid in the urine compared to other patient groups [23]. Finally, iterative longitudinal system biology studies of *P. cynomolgi* infection in rhesus macaques were carried out focusing on primary illness, relapse illness, and subsequent disease and immune response patterns, which yielded results comparable to the papers presented above [24]. However, all these studies focus on metabolic changes in active malaria infections diagnosed by the presence of blood stages, while our study focuses on detecting hypnozoite-induced metabolic changes in asymptomatic infection characterized by the absence of both growing liver stages and blood stages.

The comparative proof-of-concept studies between hypnozoite-forming (*P. cynomolgi*) and hypnozoite-non-forming (*P. knowlesi*) malaria parasite hepatocyte infections have been carried out in *in vitro* cultures because of the advantages such setting offers over a direct *in vivo* study. These advantages include likely higher concentrations and thus more easily detectable metabolites, better- defined environment and easier manipulation in a system that fully replicates the development of a *P. vivax* liver infection in humans [7,8,14]. Efficient cultures resulted in culture supernatants for analysis from hypnozoite-enriched cultures at the various timepoints detailed above. While three biological replicates were used for *P. cynomolgi* in vitro cultures, only one was used for *P. knowlesi*. Moreover, due to logistical reasons, the initial inoculum was different (P. cyonomolgi: 100,000 sporozoites vs P. knowlesi 50,000 sporozoites). Both issues should be considered a limitation of the study presented in this paper.

While both the ATQ and PI4Ki drugs enrich the cultures for hypnozoites by eliminating developing EEFs, they can also have an effect on the hepatocyte metabolome. In terms of metabolites, each sample (STD, ATQ, or PI4Ki) was normalized to its corresponding uninfected untreated (for STD) or drug-treated for (ATQ and PI4Ki) control, to minimize the chance that we will pick up metabolites due to the effect of drugs on the liver, rather than the presence of hypnozoites. Using two drugs that eliminate developing EEFs through different mechanisms of action makes us confident that we are looking at hypnozoite-induced metabolic changes. Thus, the first most stringent criteria for metabolite selection (see results section) were that the candidate metabolite be significantly up-regulated in both ATQ- and PI4Ki-treated samples.

Although the *in vitro* Proof-of-Concept study likely reproduces the range of total parasites expected in *in vivo* infected livers, the sensitivity in this system is artificially high. Thus, an important limitation of this setup concerns its direct translatability to the detection of metabolites in the plasma of *P. cynomogi*-infected rhesus macaques or *P. vivax* infected individuals, where the sensitivity is likely to be much lower. However, the purpose of this study was primarily to benchmark feasibility and show that it is possible to detect hypnozoite infection specific metabolites, thus direct translatability was a secondary concern at this stage.

A comparative approach was adopted in this research, which focuses on metabolites detected at time points particularly enriched in *P. cynomolgi* liver hypnozoites. Most importantly in order to achieve specificity in metabolic signatures the sine-qua non condition applied throughout is that the metabolites in the final selection be absent or detected only early and not significantly up-regulated in the *P. knowlesi* hepatocyte infection metabolome. To find a balance and avoid being overly restrictive in our selection to avoid missing important metabolites, reproducibility of metabolites’ detection across biological replicates though taken into account, was considered a secondary criterion at this point. Privileging specificity over reproducibility of detection across biological replicates led to the inclusion of metabolites that were detected multiple times and were highly significantly up-regulated in one single replicate. While there are both advantages and disadvantages to following such an approach given that we are looking for a needle in a haystack, we believe that this balanced approach is most suitable to finding relevant candidate metabolites. Overall, 19 metabolites were considered potentially hypnozoite-associated as a result of this analysis (Table 3).

A downstream analysis performed on the identified metabolites with known chemical structure, shows that among the metabolites identified according to our prioritization criteria, two metabolites, Lactic acid [25–27] and Kynurenine [28–30], were already described as being associated with blood-stage malaria-induced physio-pathological manifestations (such as severe malaria). Their identification through our priority criteria gives us confidence that we are able to identify malaria-related metabolites in the large pool of candidate metabolites detected. As the purpose of the research is to identify the presence of asymptomatic hypnozoite carriers, who do not have malaria symptoms and/or blood-stage parasites in circulation, these metabolites could be quite interesting and useful for our purposes. Further analysis of the metabolic pathways has shown that *N*-acetylglycine and *N*-acetylhistidine are associated with locally confined, but protracted (long-term) hepatic damage [31], which may be the result of the protracted presence of hypnozoites in *P. cynomolgi* liver infections.

Further potentially hypnozoite-associated metabolites have been identified, the chemical structure of which was unknown. An effort has been made to determine the identity of said metabolities and fully characterize them, whereby their chemical structure has been predicted through CE-HRMS analysis (Table 4) and will be confirmed using commercially available or *de novo* synthesized standards.

This *in vitro* Proof-of-Concept study offered the opportunity to identify meaningful potentially hypnozoite infection specific candidate metabolites specific to hypnozoite infection for follow-up. The set of hypnozoite-associated metabolites established here will first be validated in *in vivo* pre-clinical studies using the *P. cynomolgi*-rhesus macaque model. If successful, further downstream validation studies will be conducted in the field in order to obtain a final set of validated hypnozoite-associated metabolites in humans (deriving directly from hypnozoites or from changes in the liver metabolome induced by the presence of hypnozoites) that may be developed into specific diagnostics tools able to selectively identify hypnozoite-carriers and suitable for deployment in resource-poor settings.

## Supporting information

S1 Table Total EEF detected on days of sampling in *P. cynomolgi* and *P. knowlesi in vitro* liver-stage cultures. (XLSX)

S2 Table Cross-referenced compiled data of all biological and technical replicates. (XLSX)

S3 Table Potentially hypnozoite- infection specific candidate metabolites significantly up- or down- regulated in the different conditions (absent in *P. knowlesi*). (DOCX)

S4 Table Candidate metabolites significantly up- or down- regulated only in P. knowlesi infection. (DOCX)

S1 Fig Evolution of the EEF in the *in vitro* liver stage culture over time. In general, observed EEF first increase in numbers due to the growth of liver stage parasite and then decrease. The three *P. cynomolgi* liver stage biological replicates culture in standard conditions (dark blue lines) show stable or growing EEF until ± day 9, which then decrease as a result of the bursting of mature liver stages leaving only hypnozoites behind. As expected, the drug treated cultures (ATQ-treated: orange lines and PI4Ki-treated gray lines) show a steeper decline in EEF because the drugs kill growing liver stages. The single P. knowlesi biological replicate shows in both standard (yellow line) and PI4Ki drug-treated (teal line) conditions a similar profile as growing liver stages either burst (standard conditions) around day 6 or are eliminated by the drug.
(TIF)

S2 Fig Proportion of large and small EEFs over time in the different culture conditions and for each biological replicate. On day three the three biological replicates in *P. cynomolgi* and the single biological replicate in *P. knowlesi* are both mainly consisting of small EEF. In standard conditions, on day 6, ± 50% of the EEF have grown into large EEF in *P. cynomolgi*, while the rest remain small EEF (hypnozoites). In *P. knowlesi*, overall, a smaller proportion of the EEFs has grown on day 6, which is not significantly different between standard- and drug-treated culture conditions because *P. knowlesi* does not build hypnozoites (small EEF). On the other hand, the proportion of large EEF on day 6 observed in the three *P. cynomolgi* biological replicated is much smaller in drug treated conditions on day 6 than it is on day 6 in standard culture conditions. This is due to the fact, that the drugs kill growing EEFs and the culture is progressively enriched in hypnozoites. In standard conditions, on day 9, the proportion of large EEF starts declining due to the bursting of the more mature, large EEF. This decline continues and on day 11 the culture appears enriched in small EEF (hypnozoites). Drug-treated *P. cynomolgi* biological replicates on day 9 show a continued decrease in EEFs. The enrichment in hypnozoites (small EEF) peaks at day 11. In line with expectations, by day 9, the single *P. knowlesi* biological replicate is completely depleted of parasites both in the standard and in the drug-treated culture conditions.
(TIF)

S1 Data Full CE-MS/MS report on the identification of the biochemical structure of three unknown metabolites. (PPTX)
