# Supplementary material for: In vitro cultured malaria hypnozoites leave a footprint of specific metabolites
Source: PLoS Pathog. 2025 Oct 16;21(10):e1013577. doi: 10.1371/journal.ppat.1013577 (PMC12530531; doi:10.1371/journal.ppat.1013577)
Supplement: S3 Table — (DOCX) [file ppat.1013577.s003.docx]

| **Supplementary table 3.** Potentially hypnozoite- infection specific candidate metabolites significantly up- or down- regulated in the different conditions (absent in *P. knowlesi*)   \|  \| *P. cynomolgi* \| \| \| \| \| \| \| \| \| \| --- \| --- \| --- \| --- \| --- \| --- \| --- \| --- \| --- \| --- \| \| STD \| \| \| ATQ \| \| \| IP4K \| \| \| \| date \| ratio \| *p*-value \| date \| ratio \| *p*-value \| date \| ratio \| *p*-value \| \| **1st Criterion** \| \| \| \| \| \| \| \| \| \| \| *N*-Acetylglycine \| d2-6 (d6) \| 0,8 \| 0,041 \| d7-11 (d11) \| 1,3 \| 0,020 \| d2-6 (d6) \| 0,7 \| 0,040 \| \|  \|  \|  \|  \|  \|  \|  \| d7-11 (d11) \| 1,2 \| 0,025 \| \|  \|  \|  \|  \|  \|  \|  \| d5-9 (d9) \| 1,1 \| 0,009 \| \|  \|  \|  \|  \|  \|  \|  \| d7-11 (d11) \| 1,3 \| 0,018 \| \| *N*-Acetylhistidine \| d7-11 (d11) \| 1,3 \| 0,003 \| d5-9 (d9) \| 1,6 \| 0,025 \| d7-11 (d11) \| 1,5 \| 0,009 \| \|  \|  \|  \|  \| d7-11 (d11) \| 1,3 \| 0,007 \|  \|  \|  \| \| Lactic acid \| d7-11 (d11) \| 1,2 \| 0,029 \| d5-9 (d9) \| 1,3 \| 0,046 \| d2-6 (d6) \| 0,9 \| 0,031 \| \|  \|  \|  \|  \| d7-11 (d11) \| 1,2 \| 0,047 \| d2-6 (d6) \| 0,9 \| 0,023 \| \|  \|  \|  \|  \|  \|  \|  \| d7-11 (d11) \| 1,2 \| 0,004 \| \| Thiaproline \| d5-9 (d9) \| 2,3 \| 0,007 \| d5-9 (d9) \| 2,0 \| 0,014 \| d5-9 (d9) \| 2,1 \| 0,050 \| \| A_0106 \|  \|  \|  \|  \|  \|  \| d5-9 (d9) \| 1,2 \| 0,024 \| \| A_0071 \|  \|  \|  \| d7-11 (d11) \| 1,3 \| 0,048 \| d7-11 (d11) \| 1,3 \| 0,049 \| \| C_0348 \| d7-11 (d11) \| 1,9 \| 0,024 \| d5-9 (d9) \| 1,8 \| 0,033 \| d5-9 (d9) \| 1,3 \| 1,6E-04 \| \|  \|  \|  \|  \| d7-11 (d11) \| 1,7 \| 0,024 \| d7-11 (d11) \| 1,6 \| 0,010 \| \| C_0552 \| d5-9 (d9) \| 1,2 \| 0,025 \| d7-11 (d11) \| 1,2 \| 0,039 \| d7-11 (d11) \| 1,2 \| 0,029 \| \|  \| d7-11 (d11) \| 1,3 \| 0,022 \|  \|  \|  \|  \|  \|  \| \| **2nd Criterion** \| \| \| \| \| \| \| \| \| \| \| Gly-Ser \| d7-11 (d11) \| 1,1 \| 0,022 \| d2-6 (d6) \| 1,2 \| 0,023 \|  \|  \|  \| \|  \|  \|  \|  \| d5-9 (d9) \| 1,2 \| 0,031 \|  \|  \|  \| \|  \|  \|  \|  \| d2-6 (d6) \| 0,8 \| 0,042 \|  \|  \|  \| \|  \|  \|  \|  \| d5-9 (d9) \| 1,3 \| 0,015 \|  \|  \|  \| \| XC0053 \| d5-9 (d9) \| 1,3 \| 0,016 \|  \|  \|  \| d7-11 (d11) \| 1,2 \| 0,030 \| \| γ-Butyrobetaine \| d2-6 (d6) \| 0,7 \| 0,022 \|  \|  \|  \| d5-9 (d9) \| 1,2 \| 0,032 \| \|  \| d7-11 (d11) \| 1,1 \| 0,048 \|  \|  \|  \|  \|  \|  \| \| C_0206 \| d5-9 (d9) \| 1,9 \| 5,6E-04 \| d5-9 (d9) \| 1,8 \| 0,028 \|  \|  \|  \| \|  \| d7-11 (d11) \| 1,8 \| 0,013 \| d7-11 (d11) \| 2,1 \| 0,038 \|  \|  \|  \| \| A_0366 \| d7-11 (d11) \| 1,2 \| 0,034 \| d7-11 (d11) \| 1,2 \| 0,023 \|  \|  \|  \| \| **3rd Criterion** \| \| \| \| \| \| \| \| \| \| \| Kynurenine \| d2-6 (d6) \| 1,3 \| 0,029 \| d2-6 (d6) \| 1,7 \| 0,015 \|  \|  \|  \| \|  \| d5-9 (d9) \| 1,9 \| 0,042 \| d5-9 (d9) \| 1,6 \| 0,029 \|  \|  \|  \| \|  \| d2-6 (d6) \| 2,3 \| 0,008 \| d2-6 (d6) \| 1,3 \| 0,005 \|  \|  \|  \| \|  \| d2-6 (d6) \| 1,4 \| 0,018 \| d2-6 (d6) \| 2,0 \| 0,001 \|  \|  \|  \| \|  \| d2-6 (d6) \| 2,4 \| 0,004 \| d5-9 (d9) \| 1,8 \| 0,035 \|  \|  \|  \| \|  \| d7-11 (d11) \| 0,8 \| 0,034 \| d2-6 (d6) \| 1,9 \| 0,028 \|  \|  \|  \| \|  \|  \|  \|  \| d5-9 (d9) \| 1,3 \| 0,013 \|  \|  \|  \| \| C_0371 \| d2-6 (d6) \| 1,1 \| 0,009 \|  \|  \|  \| d7-11 (d11) \| 1,1 \| 0,012 \| \|  \|  \|  \|  \|  \|  \|  \| d7-11 (d11) \| 1,1 \| 0,002 \| \| C_0159 \| d7-11 (d11) \| 1,5 \| 0,025 \|  \|  \|  \| d5-9 (d9) \| 0,8 \| 0,030 \| \| C_0254 \| d5-9 (d9) \| 1,1 \| 0,044 \| d5-9 (d9) \| 1,2 \| 0,017 \|  \|  \|  \| \|  \| d7-11 (d11) \| 1,1 \| 0,005 \|  \|  \|  \|  \|  \|  \| \| C_0488 \| d7-11 (d11) \| 0,7 \| 0,017 \|  \|  \|  \| d5-9 (d9) \| 0,7 \| 0,012 \| \| C_0354 \| d7-11 (d11) \| 0,9 \| 0,037 \|  \|  \|  \| d5-9 (d9) \| 1,4 \| 0,036 \| |
| --- | --- | --- | --- | --- | --- | --- | --- | --- | --- | --- | --- | --- | --- | --- | --- | --- | --- | --- | --- | --- | --- | --- | --- | --- | --- | --- | --- | --- | --- | --- | --- | --- | --- | --- | --- | --- | --- | --- | --- | --- | --- | --- | --- | --- | --- | --- | --- | --- | --- | --- | --- | --- | --- | --- | --- | --- | --- | --- | --- | --- | --- | --- | --- | --- | --- | --- | --- | --- | --- | --- | --- | --- | --- | --- | --- | --- | --- | --- | --- | --- | --- | --- | --- | --- | --- | --- | --- | --- | --- | --- | --- | --- | --- | --- | --- | --- | --- | --- | --- | --- | --- | --- | --- | --- | --- | --- | --- | --- | --- | --- | --- | --- | --- | --- | --- | --- | --- | --- | --- | --- | --- | --- | --- | --- | --- | --- | --- | --- | --- | --- | --- | --- | --- | --- | --- | --- | --- | --- | --- | --- | --- | --- | --- | --- | --- | --- | --- | --- | --- | --- | --- | --- | --- | --- | --- | --- | --- | --- | --- | --- | --- | --- | --- | --- | --- | --- | --- | --- | --- | --- | --- | --- | --- | --- | --- | --- | --- | --- | --- | --- | --- | --- | --- | --- | --- | --- | --- | --- | --- | --- | --- | --- | --- | --- | --- | --- | --- | --- | --- | --- | --- | --- | --- | --- | --- | --- | --- | --- | --- | --- | --- | --- | --- | --- | --- | --- | --- | --- | --- | --- | --- | --- | --- | --- | --- | --- | --- | --- | --- | --- | --- | --- | --- | --- | --- | --- | --- | --- | --- | --- | --- | --- | --- | --- | --- | --- | --- | --- | --- | --- | --- | --- | --- | --- | --- | --- | --- | --- | --- | --- | --- | --- | --- | --- | --- | --- | --- | --- | --- | --- | --- | --- | --- | --- | --- | --- | --- | --- | --- | --- | --- | --- | --- | --- | --- | --- | --- | --- | --- | --- | --- | --- | --- | --- | --- | --- | --- | --- | --- | --- | --- | --- | --- | --- | --- | --- | --- | --- | --- | --- | --- | --- | --- | --- | --- | --- | --- | --- | --- | --- | --- | --- | --- | --- | --- | --- | --- | --- | --- | --- | --- | --- | --- | --- | --- | --- | --- | --- | --- | --- | --- | --- | --- | --- | --- | --- | --- | --- | --- | --- | --- | --- | --- | --- | --- | --- | --- | --- | --- | --- | --- | --- | --- | --- | --- | --- | --- | --- | --- | --- | --- | --- | --- | --- | --- | --- | --- | --- | --- | --- | --- | --- | --- | --- | --- | --- | --- | --- | --- | --- | --- | --- | --- | --- | --- | --- | --- | --- | --- | --- | --- | --- | --- | --- | --- | --- | --- | --- | --- | --- | --- | --- | --- | --- | --- | --- | --- | --- | --- | --- | --- | --- | --- | --- | --- | --- | --- | --- | --- | --- | --- | --- | --- | --- | --- | --- | --- | --- | --- | --- | --- | --- | --- | --- | --- | --- | --- | --- | --- | --- | --- | --- | --- | --- | --- | --- | --- | --- |
