# Supplementary material for: In vitro cultured malaria hypnozoites leave a footprint of specific metabolites
Source: PLoS Pathog. 2025 Oct 16;21(10):e1013577. doi: 10.1371/journal.ppat.1013577 (PMC12530531; doi:10.1371/journal.ppat.1013577)
Supplement: S4 Table — (DOCX) [file ppat.1013577.s004.docx]

**Supplementary table 4.** Candidate metabolites significantly up- or down- regulated only in P. knowlesi infection

|  | *P. knowlesi* | | | | | |
| --- | --- | --- | --- | --- | --- | --- |
|  | STD | | | IP4K | | |
|  | date | ratio | *p*-value | date | ratio | *p*-value |
| 2-Hydroxy-4-methylvaleric acid | d5-9 (d9) | 1,3 | 0,002 |  |  |  |
| 2-Hydroxybutyric acid | d2-6 (d6) | 0,9 | 0,018 |  |  |  |
| 8-Hydroxyoctanoic acid-1 2-Hydroxyoctanoic acid | d5-9 (d9) | 1,2 | 0,046 |  |  |  |
| Acetoacetic acid | d2-6 (d6) | 0,8 | 0,038 |  |  |  |
| Ala-Ser or Thr-Gly | d2-6 (d6) | 1,4 | 0,043 |  |  |  |
|  | d5-9 (d9) | 1,5 | 0,003 |  |  |  |
| Caffeine | d5-9 (d9) | 1,3 | 0,009 |  |  |  |
| Dihydroxyacetone phosphate | d5-9 (d9) | 2,3 | 0,012 |  |  |  |
| Fumaric acid | d5-9 (d9) | 1,2 | 0,047 |  |  |  |
| Octanoic acid |  |  |  | d5-9 (d9) | 1,4 | 0,022 |
| Pipecolic acid |  |  |  | d5-9 (d9) | 1,4 | 0,022 |
| XC0008 | d2-6 (d6) | 0,6 | 0,034 |  |  |  |
