# Supplementary figures and images for: In vitro cultured malaria hypnozoites leave a footprint of specific metabolites
Source: PLoS Pathog. 2025 Oct 16;21(10):e1013577. doi: 10.1371/journal.ppat.1013577 (PMC12530531; doi:10.1371/journal.ppat.1013577)

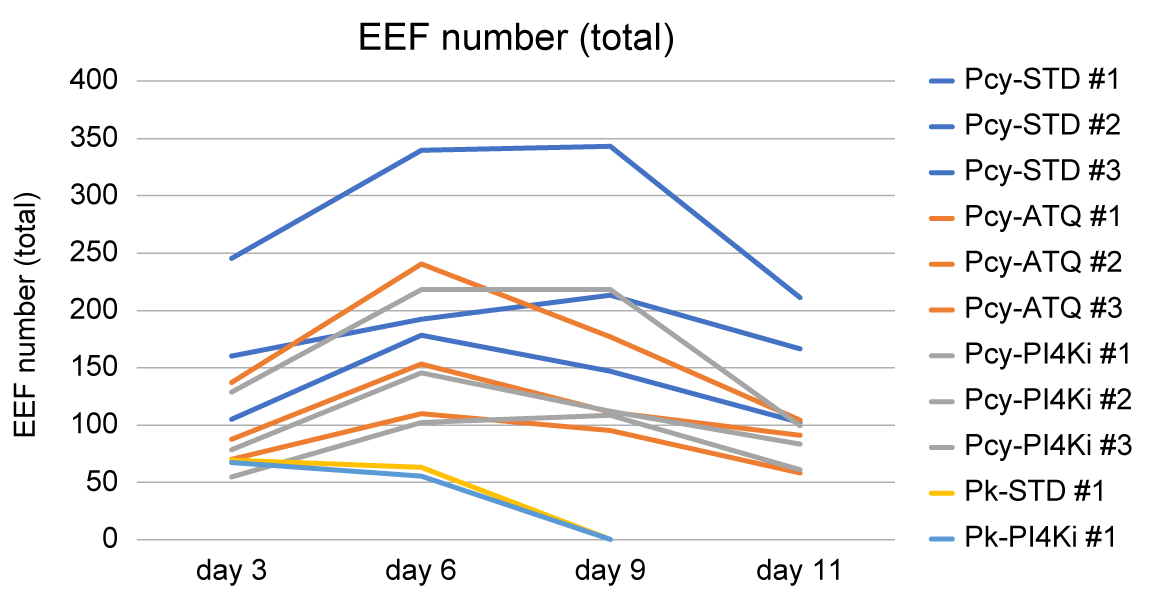

Supplement: S1 Fig — In general, observed EEF first increase in numbers due to the growth of liver stage parasite and then decrease. The three P. cynomolgi liver stage biological replicates culture in standard conditions (dark blue lines) show stable or growing EEF until ± day 9, which then decrease as a result of the bursting of mature liver stages leaving only hypnozoites behind. As expected, the drug treated cultures (ATQ-treated: orange lines and PI4Ki-treated gray lines) show a steeper decline in EEF because the drugs kill growing liver stages. The single P. knowlesi biological replicate shows in both standard (yellow line) and PI4Ki drug-treated (teal line) conditions a similar profile as growing liver stages either burst (standard conditions) around day 6 or are eliminated by the drug. (TIF) [file ppat.1013577.s005.tif]

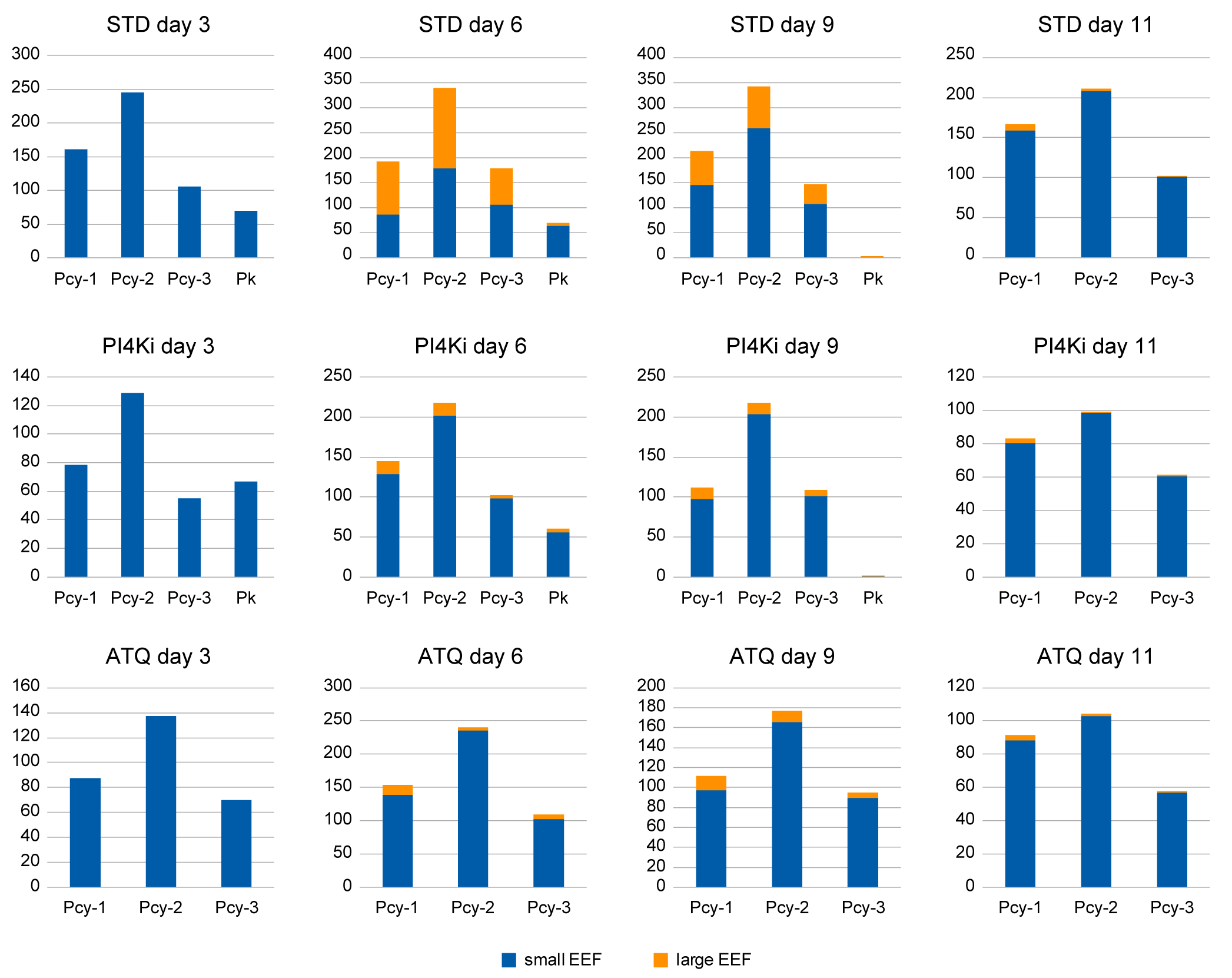

Supplement: S2 Fig — On day three the three biological replicates in P. cynomolgi and the single biological replicate in P. knowlesi are both mainly consisting of small EEF. In standard conditions, on day 6, ± 50% of the EEF have grown into large EEF in P. cynomolgi, while the rest remain small EEF (hypnozoites). In P. knowlesi, overall, a smaller proportion of the EEFs has grown on day 6, which is not significantly different between standard- and drug-treated culture conditions because P. knowlesi does not build hypnozoites (small EEF). On the other hand, the proportion of large EEF on day 6 observed in the three P. cynomolgi biological replicated is much smaller in drug treated conditions on day 6 than it is on day 6 in standard culture conditions. This is due to the fact, that the drugs kill growing EEFs and the culture is progressively enriched in hypnozoites. In standard conditions, on day 9, the proportion of large EEF starts declining due to the bursting of the more mature, large EEF. This decline continues and on day 11 the culture appears enriched in small EEF (hypnozoites). Drug-treated P. cynomolgi biological replicates on day 9 show a continued decrease in EEFs. The enrichment in hypnozoites (small EEF) peaks at day 11. In line with expectations, by day 9, the single P. knowlesi biological replicate is completely depleted of parasites both in the standard and in the drug-treated culture conditions. (TIF) [file ppat.1013577.s006.tif]
