# Supplementary material for: In vitro cultured malaria hypnozoites leave a footprint of specific metabolites
Source: PLoS Pathog. 2025 Oct 16;21(10):e1013577. doi: 10.1371/journal.ppat.1013577 (PMC12530531; doi:10.1371/journal.ppat.1013577)
Supplement: S1 Data — (PPTX) [file ppat.1013577.s007.pptx]

## Slide 1
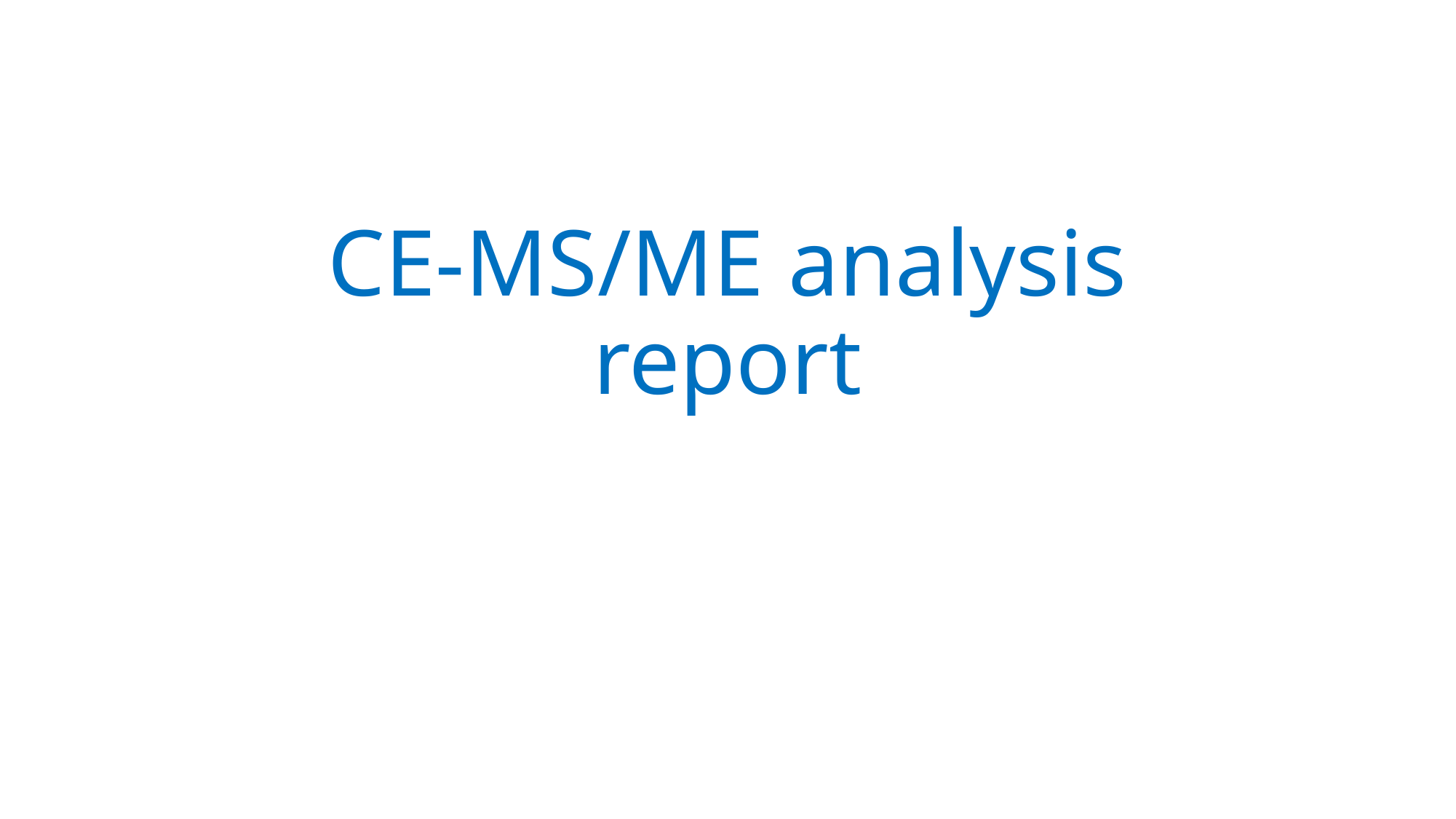

# CE-MS/ME analysis report

## Slide 2
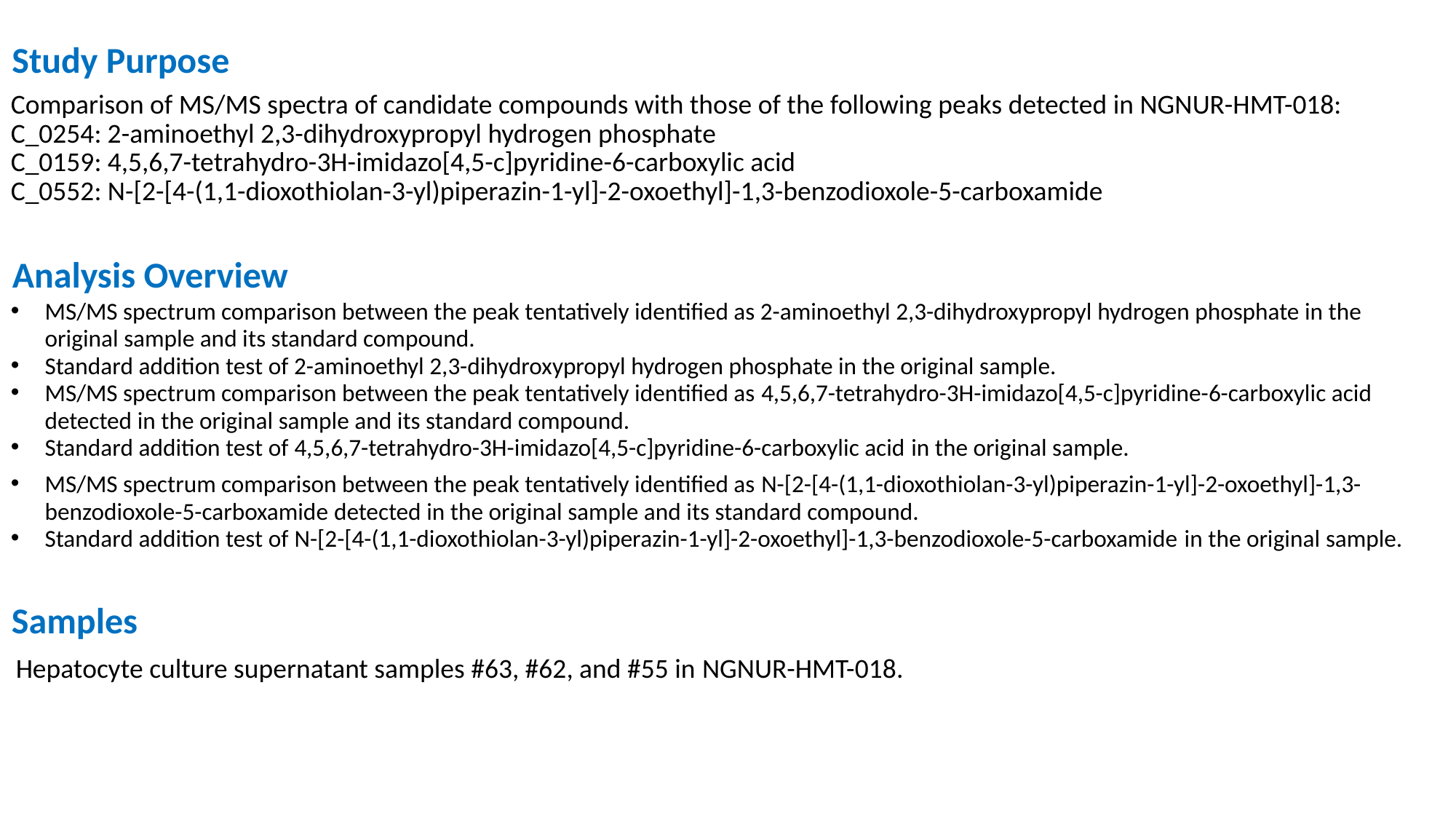

Study Purpose
Comparison of MS/MS spectra of candidate compounds with those of the following peaks detected in NGNUR-HMT-018:
C_0254: 2-aminoethyl 2,3-dihydroxypropyl hydrogen phosphate
C_0159: 4,5,6,7-tetrahydro-3H-imidazo[4,5-c]pyridine-6-carboxylic acid
C_0552: N-[2-[4-(1,1-dioxothiolan-3-yl)piperazin-1-yl]-2-oxoethyl]-1,3-benzodioxole-5-carboxamide
Analysis Overview
MS/MS spectrum comparison between the peak tentatively identified as 2-aminoethyl 2,3-dihydroxypropyl hydrogen phosphate in the original sample and its standard compound.
Standard addition test of 2-aminoethyl 2,3-dihydroxypropyl hydrogen phosphate in the original sample.
MS/MS spectrum comparison between the peak tentatively identified as 4,5,6,7-tetrahydro-3H-imidazo[4,5-c]pyridine-6-carboxylic acid detected in the original sample and its standard compound.
Standard addition test of 4,5,6,7-tetrahydro-3H-imidazo[4,5-c]pyridine-6-carboxylic acid in the original sample.
MS/MS spectrum comparison between the peak tentatively identified as N-[2-[4-(1,1-dioxothiolan-3-yl)piperazin-1-yl]-2-oxoethyl]-1,3-benzodioxole-5-carboxamide detected in the original sample and its standard compound.
Standard addition test of N-[2-[4-(1,1-dioxothiolan-3-yl)piperazin-1-yl]-2-oxoethyl]-1,3-benzodioxole-5-carboxamide in the original sample.
Samples
Hepatocyte culture supernatant samples #63, #62, and #55 in NGNUR-HMT-018.

## Slide 3
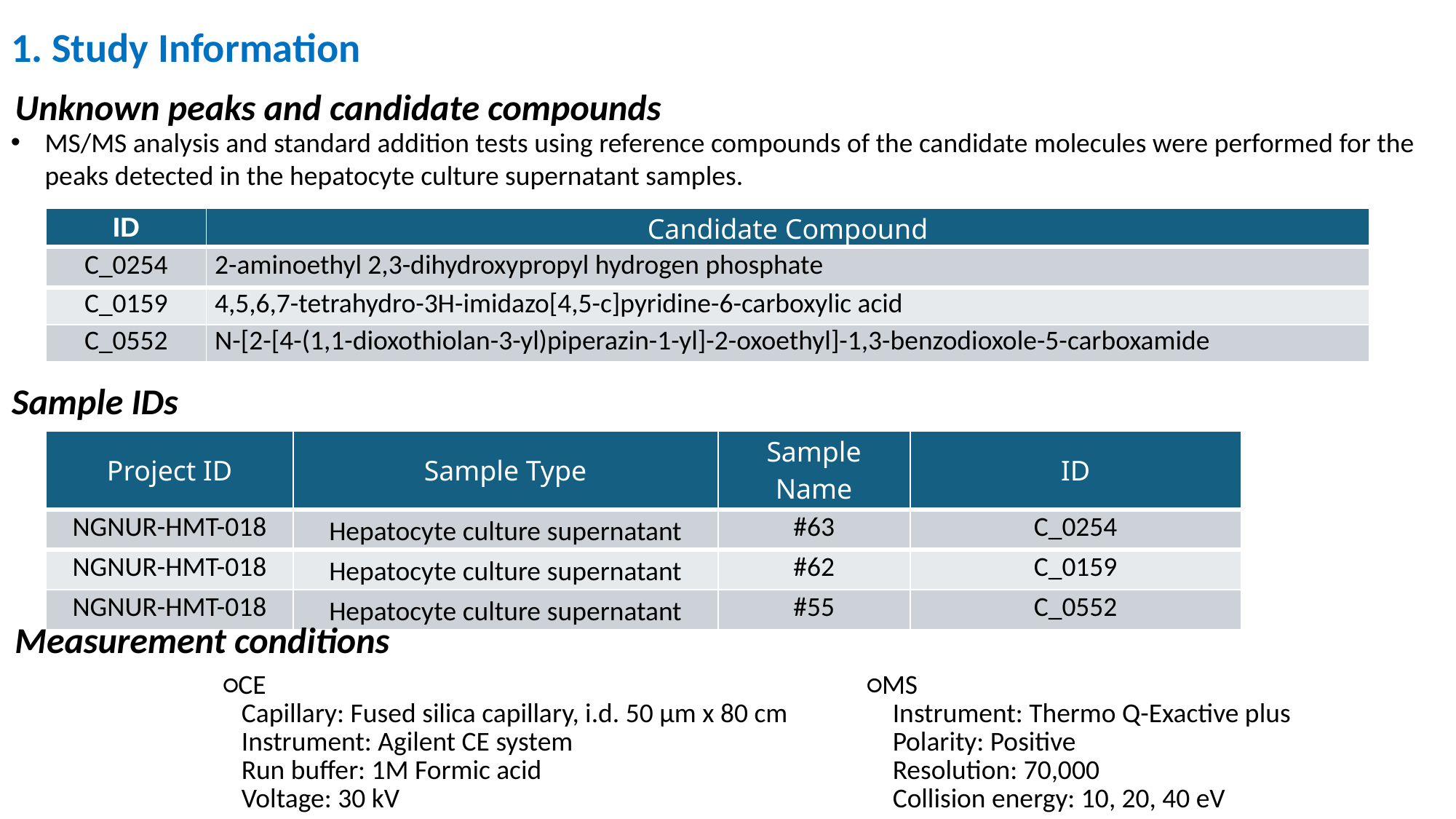

# 1. Study Information
Unknown peaks and candidate compounds
MS/MS analysis and standard addition tests using reference compounds of the candidate molecules were performed for the peaks detected in the hepatocyte culture supernatant samples.
| ID | Candidate Compound |
| --- | --- |
| C\_0254 | 2-aminoethyl 2,3-dihydroxypropyl hydrogen phosphate |
| C\_0159 | 4,5,6,7-tetrahydro-3H-imidazo[4,5-c]pyridine-6-carboxylic acid |
| C\_0552 | N-[2-[4-(1,1-dioxothiolan-3-yl)piperazin-1-yl]-2-oxoethyl]-1,3-benzodioxole-5-carboxamide |
Sample IDs
| Project ID | Sample Type | Sample Name | ID |
| --- | --- | --- | --- |
| NGNUR-HMT-018 | Hepatocyte culture supernatant | #63 | C\_0254 |
| NGNUR-HMT-018 | Hepatocyte culture supernatant | #62 | C\_0159 |
| NGNUR-HMT-018 | Hepatocyte culture supernatant | #55 | C\_0552 |
Measurement conditions
 ○MS
Instrument: Thermo Q-Exactive plus
Polarity: Positive
Resolution: 70,000
Collision energy: 10, 20, 40 eV
○CE
Capillary: Fused silica capillary, i.d. 50 µm x 80 cm
Instrument: Agilent CE system
Run buffer: 1M Formic acid
Voltage: 30 kV

## Slide 4
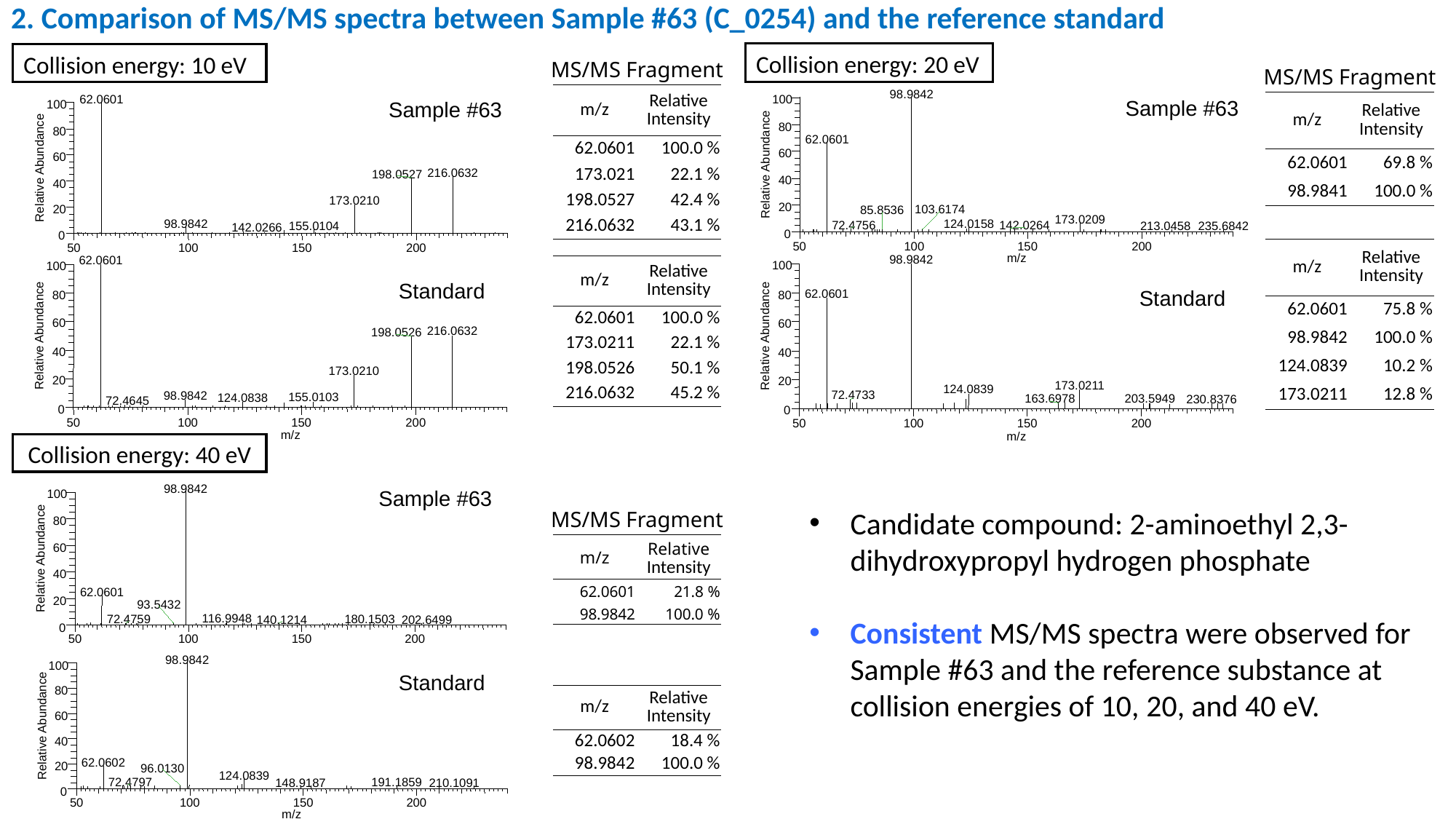

# 2. Comparison of MS/MS spectra between Sample #63 (C_0254) and the reference standard
MS/MS Fragment
Collision energy: 20 eV
Collision energy: 10 eV
MS/MS Fragment
98.9842
100
80
62.0601
60
Relative Abundance
40
20
103.6174
85.8536
173.0209
124.0158
72.4756
142.0264
213.0458
235.6842
0
50
100
150
200
m/z
62.0601
100
80
60
Relative Abundance
216.0632
198.0527
40
173.0210
20
98.9842
155.0104
142.0266
0
50
100
150
200
Sample #63
Sample #63
| m/z | Relative Intensity |
| --- | --- |
| 62.0601 | 100.0 % |
| 173.021 | 22.1 % |
| 198.0527 | 42.4 % |
| 216.0632 | 43.1 % |
| m/z | Relative Intensity |
| --- | --- |
| 62.0601 | 69.8 % |
| 98.9841 | 100.0 % |
98.9842
100
62.0601
80
60
Relative Abundance
40
20
173.0211
124.0839
72.4733
163.6978
203.5949
230.8376
0
50
100
150
200
m/z
62.0601
100
80
60
216.0632
198.0526
Relative Abundance
40
173.0210
20
98.9842
155.0103
124.0838
72.4645
0
50
100
150
200
m/z
| m/z | Relative Intensity |
| --- | --- |
| 62.0601 | 75.8 % |
| 98.9842 | 100.0 % |
| 124.0839 | 10.2 % |
| 173.0211 | 12.8 % |
| m/z | Relative Intensity |
| --- | --- |
| 62.0601 | 100.0 % |
| 173.0211 | 22.1 % |
| 198.0526 | 50.1 % |
| 216.0632 | 45.2 % |
Standard
Standard
Collision energy: 40 eV
98.9842
100
80
60
Relative Abundance
40
62.0601
20
93.5432
116.9948
72.4759
180.1503
140.1214
202.6499
0
50
100
150
200
Sample #63
MS/MS Fragment
Candidate compound: 2-aminoethyl 2,3-dihydroxypropyl hydrogen phosphate
Consistent MS/MS spectra were observed for Sample #63 and the reference substance at collision energies of 10, 20, and 40 eV.
| m/z | Relative Intensity |
| --- | --- |
| 62.0601 | 21.8 % |
| 98.9842 | 100.0 % |
98.9842
100
80
60
Relative Abundance
40
62.0602
20
96.0130
124.0839
72.4797
191.1859
148.9187
210.1091
0
50
100
150
200
m/z
Standard
| m/z | Relative Intensity |
| --- | --- |
| 62.0602 | 18.4 % |
| 98.9842 | 100.0 % |

## Slide 5
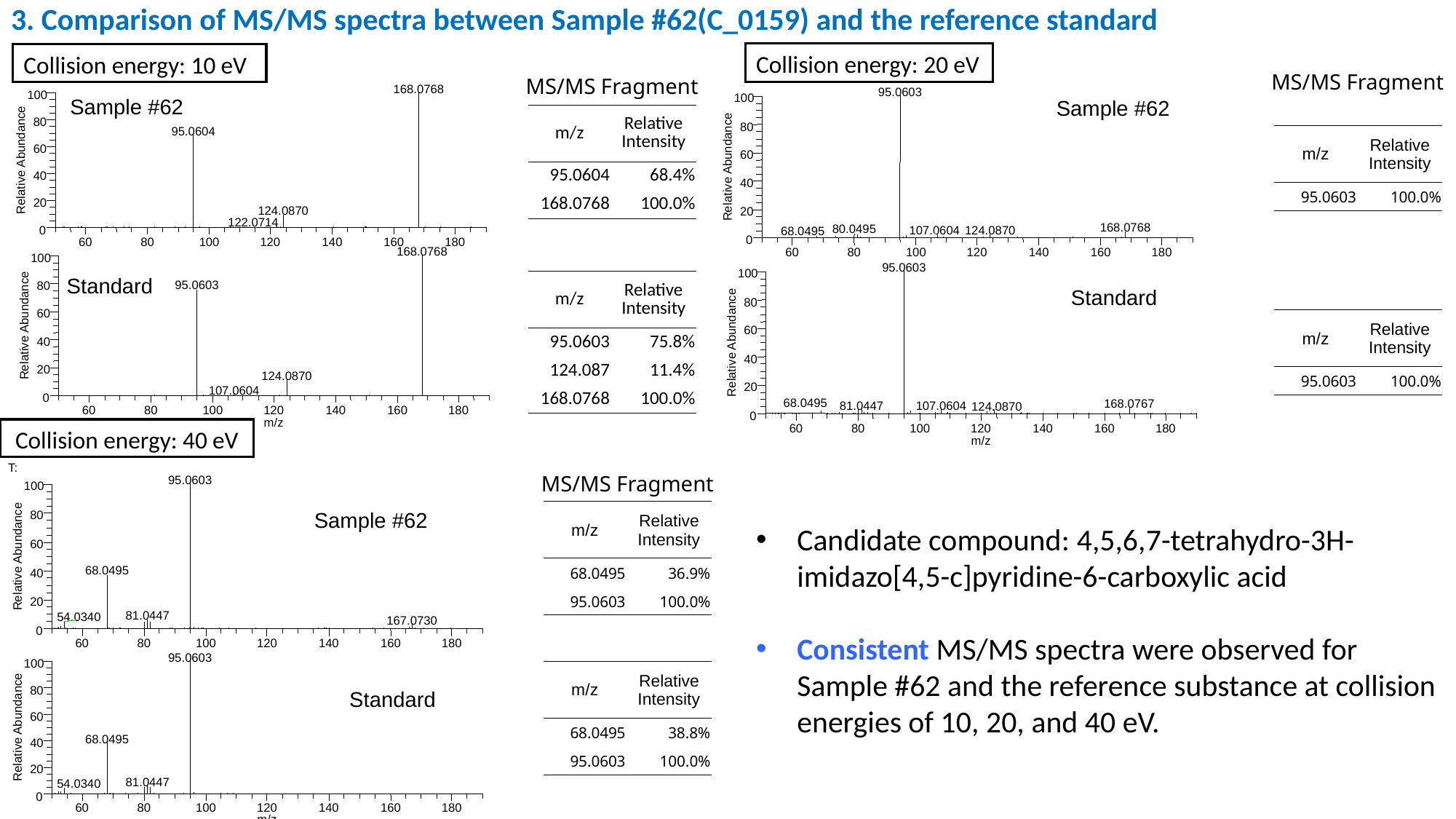

# 3. Comparison of MS/MS spectra between Sample #62(C_0159) and the reference standard
Collision energy: 20 eV
Collision energy: 10 eV
MS/MS Fragment
168.0768
100
80
95.0604
60
Relative Abundance
40
20
124.0870
122.0714
0
60
80
100
120
140
160
180
MS/MS Fragment
95.0603
100
80
60
Relative Abundance
40
20
168.0768
80.0495
107.0604
124.0870
68.0495
0
60
80
100
120
140
160
180
Sample #62
Sample #62
| m/z | Relative Intensity |
| --- | --- |
| 95.0604 | 68.4% |
| 168.0768 | 100.0% |
| m/z | Relative Intensity |
| --- | --- |
| 95.0603 | 100.0% |
168.0768
100
95.0603
80
60
Relative Abundance
40
20
124.0870
107.0604
0
60
80
100
120
140
160
180
m/z
95.0603
100
80
60
Relative Abundance
40
20
68.0495
168.0767
81.0447
107.0604
124.0870
0
60
80
100
120
140
160
180
m/z
Standard
| m/z | Relative Intensity |
| --- | --- |
| 95.0603 | 75.8% |
| 124.087 | 11.4% |
| 168.0768 | 100.0% |
Standard
| m/z | Relative Intensity |
| --- | --- |
| 95.0603 | 100.0% |
Collision energy: 40 eV
T:
95.0603
100
80
60
Relative Abundance
68.0495
40
20
81.0447
54.0340
167.0730
0
60
80
100
120
140
160
180
MS/MS Fragment
Sample #62
| m/z | Relative Intensity |
| --- | --- |
| 68.0495 | 36.9% |
| 95.0603 | 100.0% |
Candidate compound: 4,5,6,7-tetrahydro-3H-imidazo[4,5-c]pyridine-6-carboxylic acid
Consistent MS/MS spectra were observed for Sample #62 and the reference substance at collision energies of 10, 20, and 40 eV.
95.0603
100
80
60
Relative Abundance
68.0495
40
20
81.0447
54.0340
0
60
80
100
120
140
160
180
m/z
| m/z | Relative Intensity |
| --- | --- |
| 68.0495 | 38.8% |
| 95.0603 | 100.0% |
Standard

## Slide 6
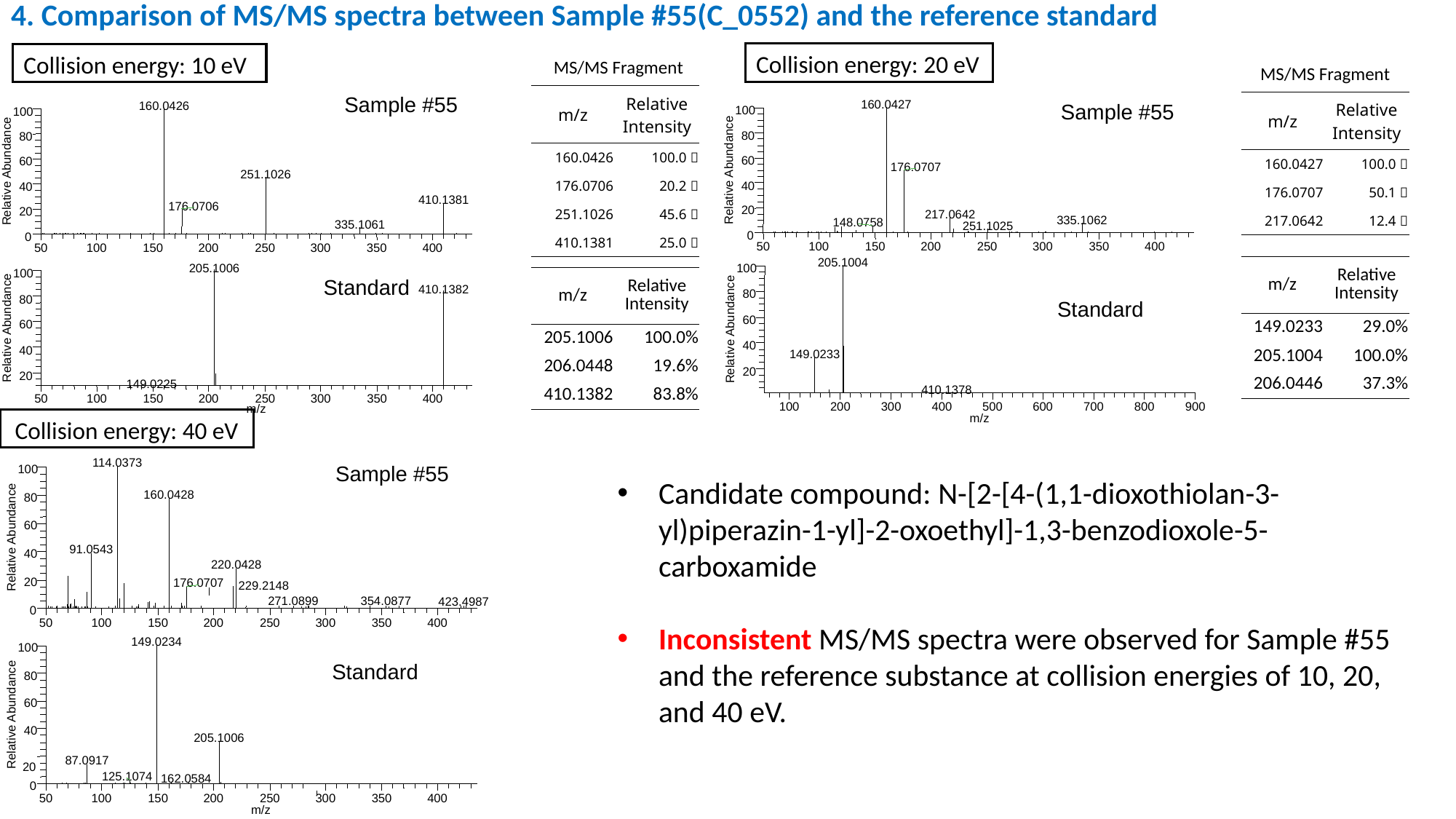

# 4. Comparison of MS/MS spectra between Sample #55(C_0552) and the reference standard
MS/MS Fragment
Collision energy: 20 eV
Collision energy: 10 eV
MS/MS Fragment
160.0427
100
80
60
176.0707
Relative Abundance
40
20
217.0642
335.1062
148.0758
251.1025
0
50
100
150
200
250
300
350
400
160.0426
100
80
60
Relative Abundance
251.1026
40
410.1381
176.0706
20
335.1061
0
50
100
150
200
250
300
350
400
Sample #55
| m/z | Relative Intensity |
| --- | --- |
| 160.0426 | 100.0％ |
| 176.0706 | 20.2％ |
| 251.1026 | 45.6％ |
| 410.1381 | 25.0％ |
Sample #55
| m/z | Relative Intensity |
| --- | --- |
| 160.0427 | 100.0％ |
| 176.0707 | 50.1％ |
| 217.0642 | 12.4％ |
205.1004
100
80
60
Relative Abundance
40
149.0233
20
410.1378
100
200
300
400
500
600
700
800
900
m/z
205.1006
100
410.1382
80
60
Relative Abundance
40
20
149.0225
50
100
150
200
250
300
350
400
m/z
| m/z | Relative Intensity |
| --- | --- |
| 149.0233 | 29.0% |
| 205.1004 | 100.0% |
| 206.0446 | 37.3% |
Standard
| m/z | Relative Intensity |
| --- | --- |
| 205.1006 | 100.0% |
| 206.0448 | 19.6% |
| 410.1382 | 83.8% |
Standard
Collision energy: 40 eV
114.0373
100
160.0428
80
60
Relative Abundance
91.0543
40
220.0428
20
176.0707
229.2148
271.0899
354.0877
423.4987
0
50
100
150
200
250
300
350
400
Sample #55
Candidate compound: N-[2-[4-(1,1-dioxothiolan-3-yl)piperazin-1-yl]-2-oxoethyl]-1,3-benzodioxole-5-carboxamide
Inconsistent MS/MS spectra were observed for Sample #55 and the reference substance at collision energies of 10, 20, and 40 eV.
149.0234
100
80
60
Relative Abundance
40
205.1006
87.0917
20
125.1074
162.0584
0
50
100
150
200
250
300
350
400
m/z
Standard

## Slide 7
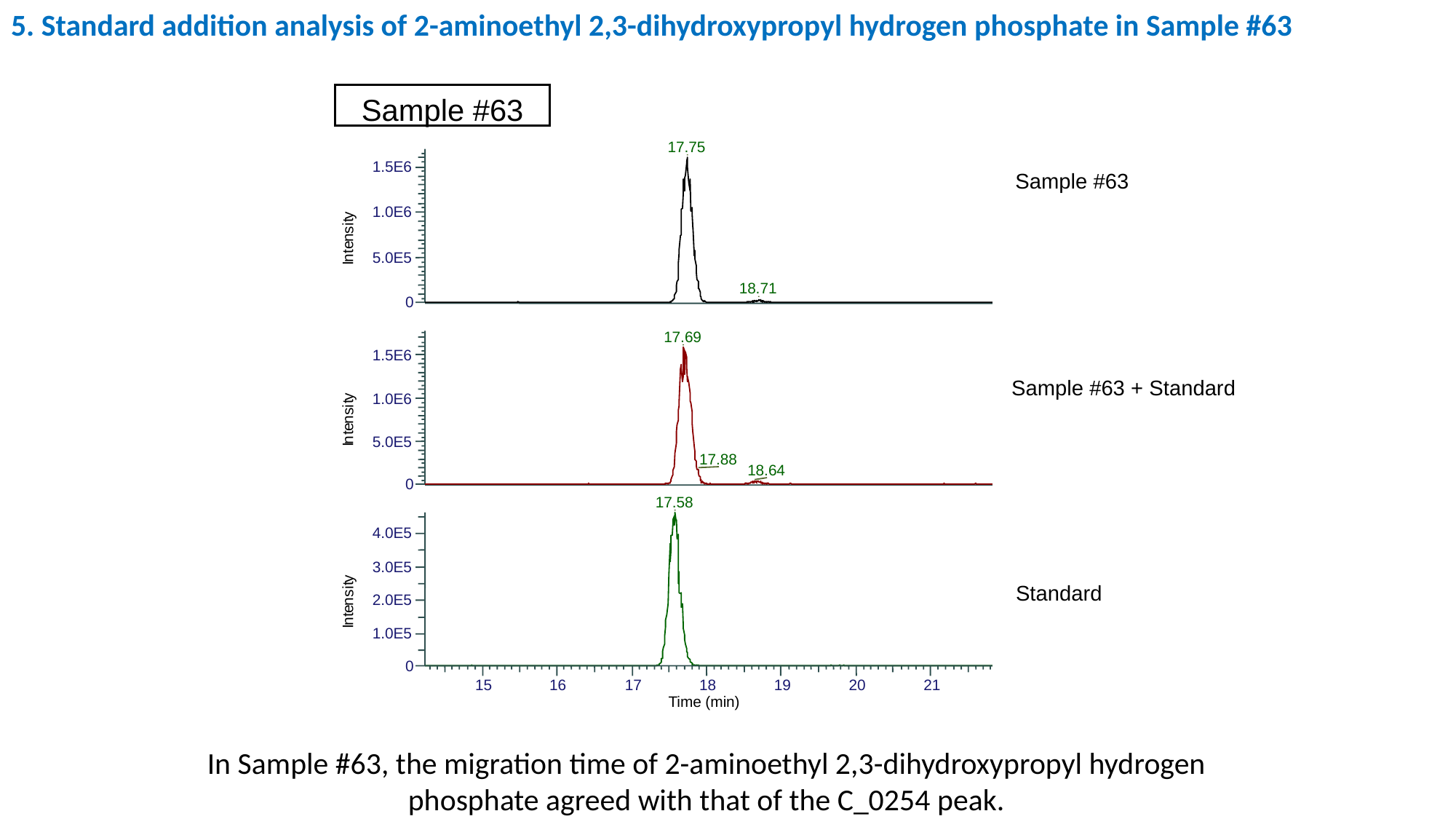

# 5. Standard addition analysis of 2-aminoethyl 2,3-dihydroxypropyl hydrogen phosphate in Sample #63
Sample #63
17.75
1.5E6
1.0E6
y
t
i
s
n
e
t
n
5.0E5
I
18.71
0
17.69
1.5E6
y
1.0E6
t
i
s
n
e
t
n
5.0E5
I
17.88
18.64
0
17.58
4.0E5
3.0E5
y
t
i
s
2.0E5
n
e
t
n
I
1.0E5
0
15
16
17
18
19
20
21
Time (min)
Sample #63
Sample #63 + Standard
Standard
In Sample #63, the migration time of 2-aminoethyl 2,3-dihydroxypropyl hydrogen phosphate agreed with that of the C_0254 peak.

## Slide 8
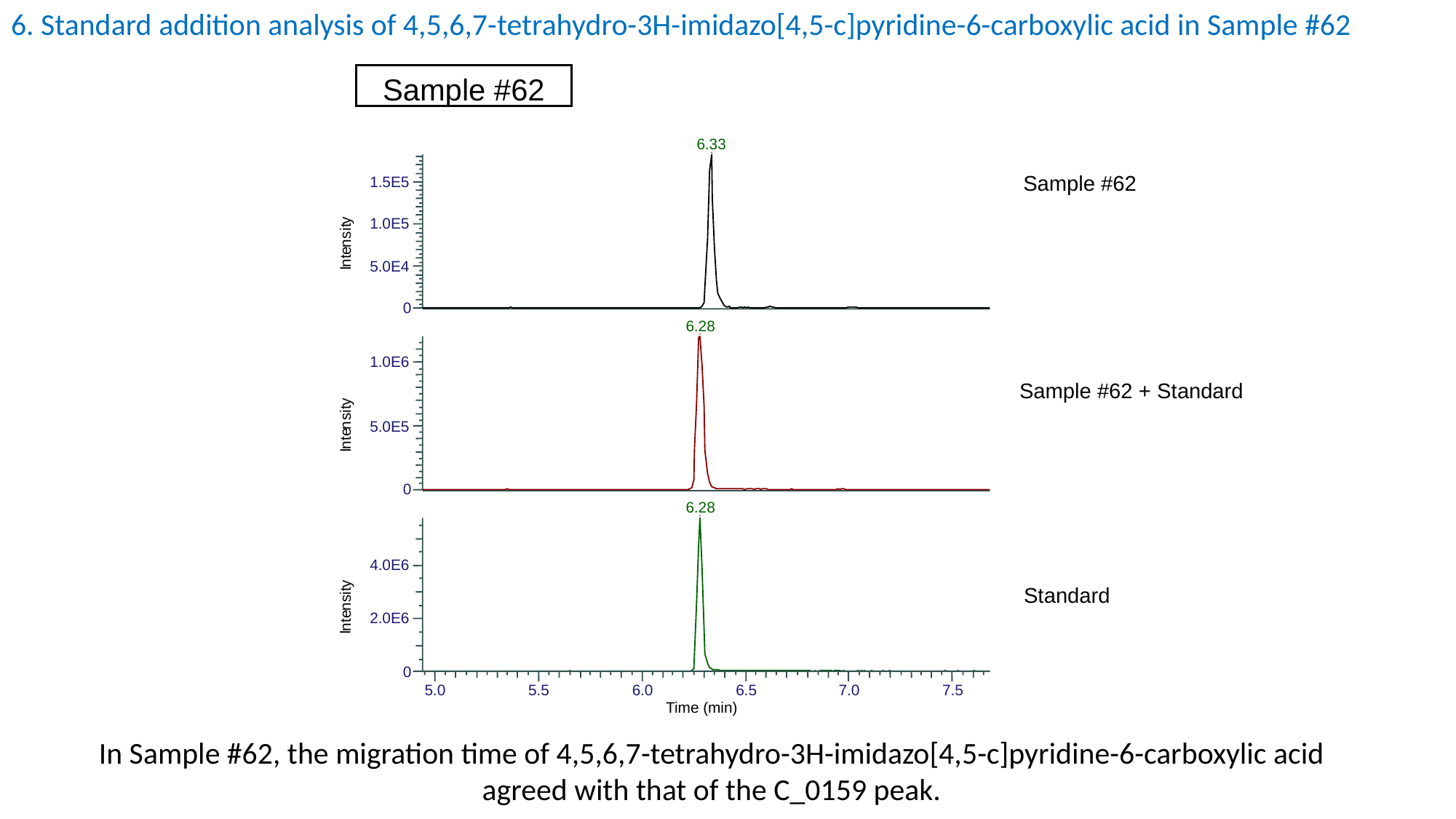

# 6. Standard addition analysis of 4,5,6,7-tetrahydro-3H-imidazo[4,5-c]pyridine-6-carboxylic acid in Sample #62
Sample #62
6.33
1.5E5
y
1.0E5
t
i
s
n
e
t
n
5.0E4
I
0
6.28
1.0E6
y
t
i
s
n
5.0E5
e
t
n
I
0
6.28
4.0E6
y
t
i
s
n
e
2.0E6
t
n
I
0
5.0
5.5
6.0
6.5
7.0
7.5
Time (min)
Sample #62
Sample #62 + Standard
Standard
In Sample #62, the migration time of 4,5,6,7-tetrahydro-3H-imidazo[4,5-c]pyridine-6-carboxylic acid agreed with that of the C_0159 peak.

## Slide 9
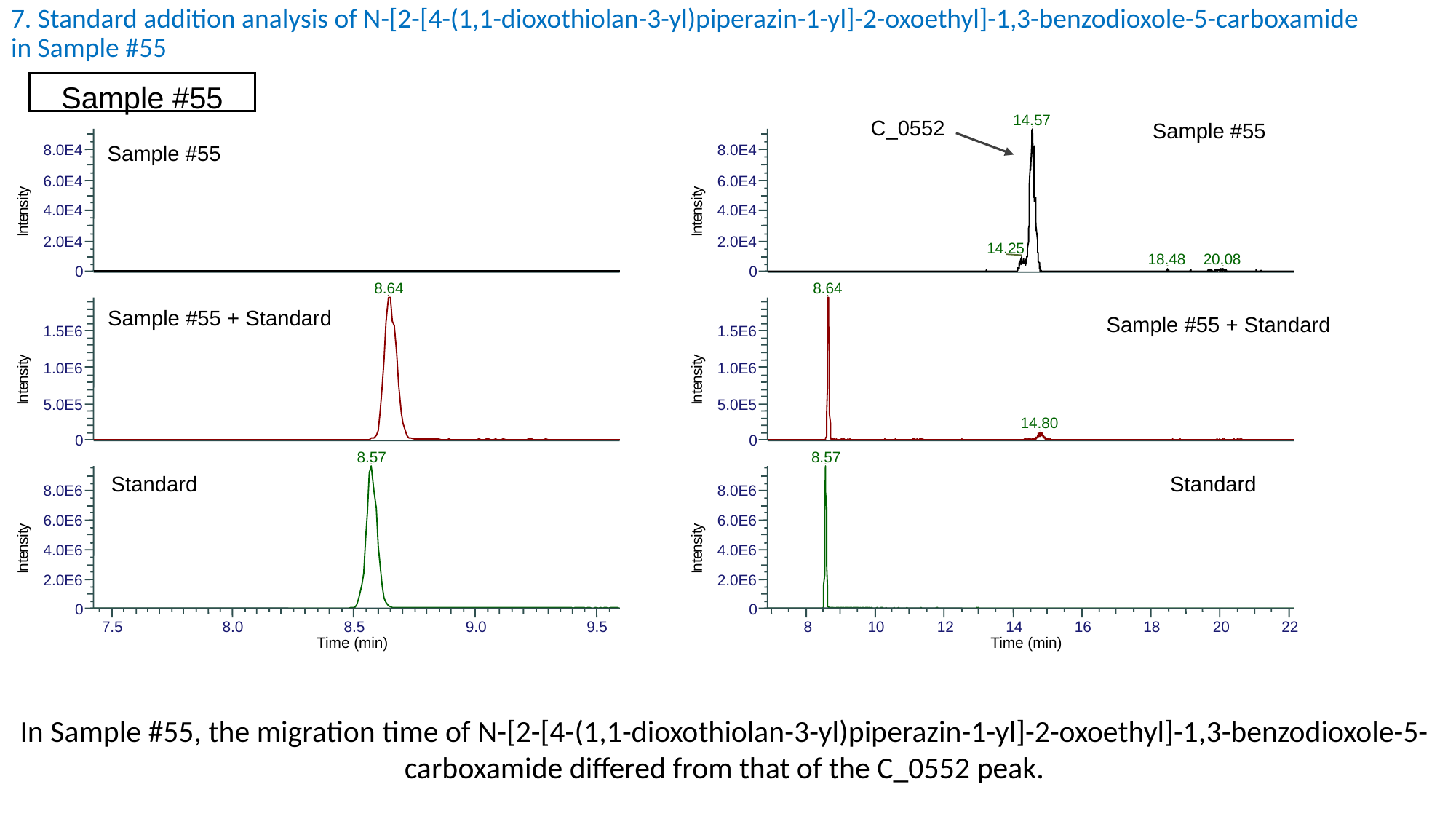

# 7. Standard addition analysis of N-[2-[4-(1,1-dioxothiolan-3-yl)piperazin-1-yl]-2-oxoethyl]-1,3-benzodioxole-5-carboxamide in Sample #55
Sample #55
8.0E4
6.0E4
y
t
i
s
4.0E4
n
e
t
n
I
2.0E4
0
8.64
1.5E6
y
t
i
1.0E6
s
n
e
t
n
I
5.0E5
0
8.57
8.0E6
6.0E6
y
t
i
s
n
4.0E6
e
t
n
I
2.0E6
0
7.5
8.0
8.5
9.0
9.5
Time (min)
14.57
8.0E4
6.0E4
y
t
i
s
4.0E4
n
e
t
n
I
2.0E4
14.25
18.48
20.08
0
8.64
1.5E6
y
t
i
1.0E6
s
n
e
t
n
I
5.0E5
14.80
0
8.57
8.0E6
6.0E6
y
t
i
s
n
4.0E6
e
t
n
I
2.0E6
0
8
10
12
14
16
18
20
22
Time (min)
C_0552
Sample #55
Sample #55
Sample #55 + Standard
Sample #55 + Standard
Standard
Standard
In Sample #55, the migration time of N-[2-[4-(1,1-dioxothiolan-3-yl)piperazin-1-yl]-2-oxoethyl]-1,3-benzodioxole-5-carboxamide differed from that of the C_0552 peak.

## Slide 10
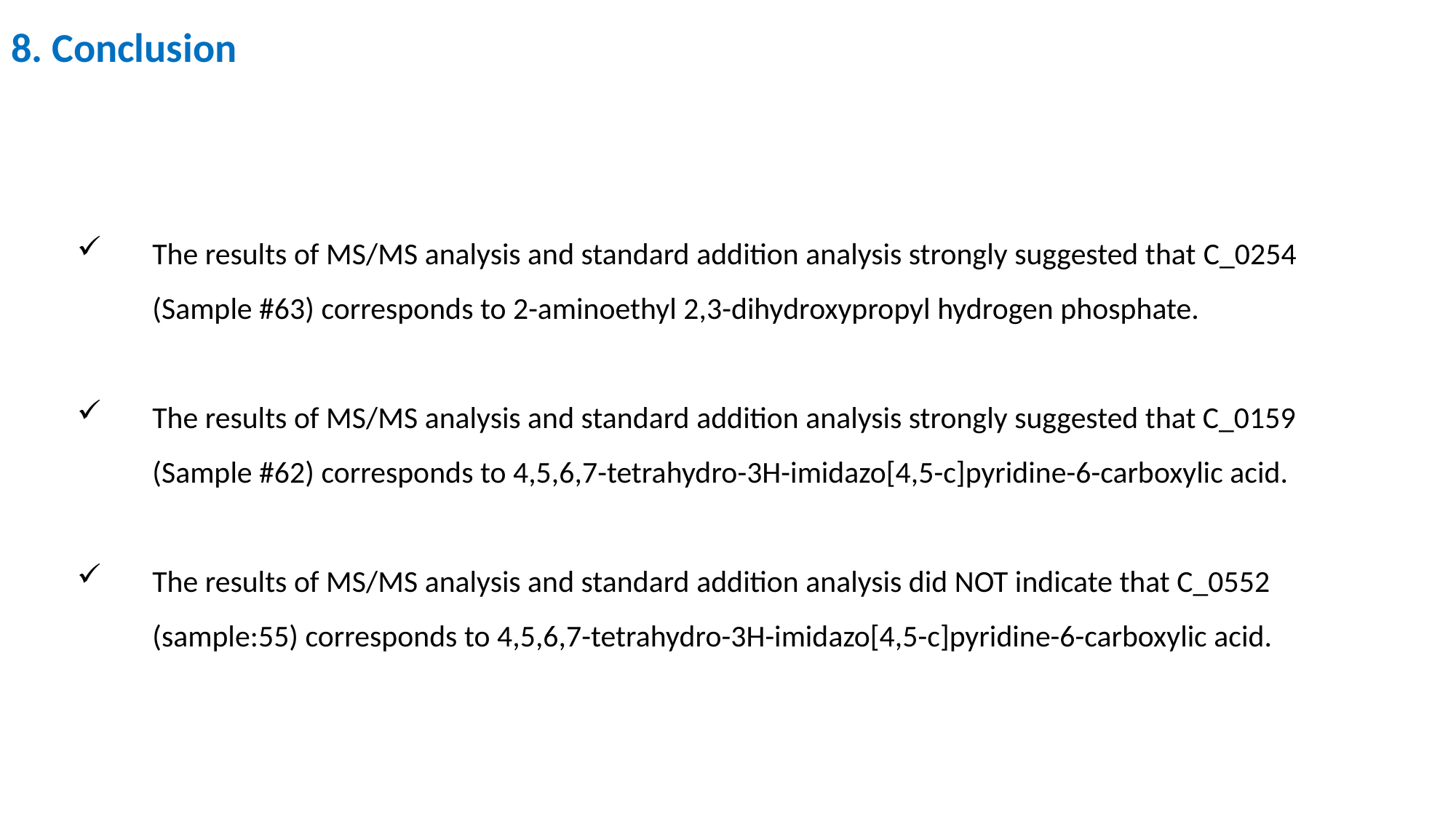

# 8. Conclusion
The results of MS/MS analysis and standard addition analysis strongly suggested that C_0254 (Sample #63) corresponds to 2-aminoethyl 2,3-dihydroxypropyl hydrogen phosphate.
The results of MS/MS analysis and standard addition analysis strongly suggested that C_0159 (Sample #62) corresponds to 4,5,6,7-tetrahydro-3H-imidazo[4,5-c]pyridine-6-carboxylic acid.
The results of MS/MS analysis and standard addition analysis did NOT indicate that C_0552 (sample:55) corresponds to 4,5,6,7-tetrahydro-3H-imidazo[4,5-c]pyridine-6-carboxylic acid.
